# Longitudinal association of cytokine-producing CMV-specific T cells with frailty in HIV-infected and -uninfected men who have sex with men

**DOI:** 10.1186/s12979-022-00270-3

**Published:** 2022-03-07

**Authors:** Weiying Zhang, Huifen Li, Jay H. Bream, Tricia L. Nilles, Sean X. Leng, Joseph B. Margolick

**Affiliations:** 1 Department of Molecular Microbiology and Immunology, Johns Hopkins Bloomberg School of Public Health, 615 N Wolfe St., Rm E5153, Baltimore, MD 21205 USA; 2 Division of Geriatric Medicine and Gerontology, Department of Medicine, Johns Hopkins School of Medicine, Baltimore, MD USA; 3 Graduate Program in Immunology, Johns Hopkins School of Medicine, Baltimore, MD USA; 4 Johns Hopkins Center on Aging and Immune Remodeling, Baltimore, MD USA

**Keywords:** CMV, CD4 T cells, CD8 T cells, Cytokine production, IFN-γ, TNF-⍺, IL-2, MACS

## Abstract

**Background:**

Chronic cytomegalovirus (CMV) infection has been postulated as a driver of chronic inflammation that has been associated with frailty and other age-related conditions in both HIV-infected (HIV+) and -uninfected (HIV-) people.

**Methods:**

To study the T cell response to CMV as a predictor of onset and maintenance of frailty, baseline CMV-specific T cell responses of 42 men (20 HIV-, 22 HIV+; 21 frail, 21 nonfrail) in the Multicenter AIDS Cohort Study (MACS) were assessed by flow cytometric analysis of cytokine production (IFN-γ, TNF-⍺, and IL-2) in response to overlapping peptide pools spanning 19 CMV open reading frames. The Fried frailty phenotype was assessed at baseline and semiannually thereafter. Times to transition into or out of frailty were compared by tertiles of percentages of cytokine-producing T cells using Kaplan-Meier estimators and the exact log-rank test.

**Results:**

Over a median follow-up of 6.5 (interquartile range: 2) years, faster onset of frailty was significantly predicted by higher (HIV- men) or lower (HIV+ men) percentages of CD4 T cells producing only IFN-γ (IFN-γ-single-producing (SP)), and by lower percentages of IFN-γ-, TNF-⍺-, and IL-2-triple-producing CD8 T cells (HIV- men). Greater maintenance of frailty was significantly predicted by lower percentages of both these T cell subsets in HIV- men, and by lower percentages of IFN-γ-SP CD4 T cells in HIV+ men. The antigenic specificity of IFN-γ-SP CD4 T cells was different between HIV- and HIV+ nonfrail men, as were the correlations between these cells and serum inflammatory markers.

**Conclusions:**

In this pilot study, percentages of CMV-specific T cells predicted the onset and maintenance of frailty in HIV- and HIV+ men. Predictive responses differed by HIV status, which may relate to differential control of CMV reactivation and inflammation by anti-CMV T cell responses.

**Supplementary Information:**

The online version contains supplementary material available at 10.1186/s12979-022-00270-3.

## Introduction

Despite effective combination antiretroviral therapy (cART), HIV-infected individuals (HIV+) are at higher risk than HIV-uninfected (HIV-) people for age-related diseases and geriatric syndromes [1, 2]. Among the latter, frailty is of particular interest considering its high prevalence in older adults [3], association with dysfunction of multiple physiological systems [4–6], and portent of poor health outcomes including death [6].

The pathophysiology of frailty is not well understood [5, 6], but chronic inflammation, as reflected in elevated circulating levels of proinflammatory markers such as interleukin-6 (IL-6), C-reactive protein (CRP), and tumor necrosis factor-α (TNF-α) [7–9], appears to be a contributing factor in both HIV- and HIV+ people. Increasing age is characterized by elevated levels of these markers, as is treated HIV infection even after effective viral suppression [7, 8]. However, the cause of chronic inflammation in these circumstances remains unknown.

One possible driver of chronic inflammation is persistent cytomegalovirus (CMV) infection. HIV- people who are CMV-seropositive have a higher prevalence of frailty than those who are -seronegative [10], and in one study older women who had detectable CMV DNA in peripheral blood monocytes had higher serum concentrations of IL-6 than those who did not [11]. Among HIV+ people, higher titers of anti-CMV IgG antibodies have been associated with subclinical cardiovascular disease [12], another condition linked to chronic inflammation [8]. In both HIV- and HIV+ people, chronic CMV infection triggers an unusually robust T cell response, with an expansion of CMV-specific T cells that often comprises 10-20%, or even more, of the circulating T cell pool [13–15]; and we found that the magnitude of this response was strongly correlated with serum concentrations of some inflammatory markers in men in the Multicenter AIDS Cohort Study (MACS) [16]. Indeed, in that study the total percentage of CD4 T cells producing IL-2 in response to a broad panel of CMV antigens predicted onset of frailty in HIV- but not HIV+ nonfrail men [16]. These observations suggest that CMV-specific T cells play an important role in the pathogenesis of chronic inflammation and frailty.

CMV-specific CD4 and CD8 T cells can produce many different combinations of cytokines [17–26], and production of different cytokines determines functionality of T cells [27] and relates to control of viral infections [28]. No studies have evaluated the relationship between polyfunctional CMV-responsive T cells and onset or maintenance of frailty. Therefore, we studied the relationship between subsets of CMV-responsive CD4 and CD8 T producing IFN-γ, TNF-α, and/or IL-2, alone or in combination, and onset of frailty in HIV- and virally suppressed HIV+ men in the MACS. Since frailty can wax and wane, especially in HIV+ men [29], we also studied the relation between CMV-responsive T cells and maintenance of frailty.

## Methods

### Study population

The MACS is a prospective cohort study, initiated in 1984, of HIV infection in men who have sex with men. Participants are seen semiannually at 4 sites across the US [30]. At each study visit, participants’ medical and behavior history are recorded, physical exam and laboratory testing are performed, and plasma, serum, and peripheral blood mononuclear cells (PBMCs) are stored [30]. Participants have been assessed for frailty via the Fried frailty phenotype (FP) at each study visit since 2007 [29]. Briefly, participants are considered frail at a given visit if they fulfill at least 3 of 5 criteria: slow walking speed, low grip strength, exhaustion, low physical activity, and weight loss. To minimize misclassification, in the present study frailty and nonfrailty at baseline were defined as expression or non-expression of the FP, respectively, at two consecutive study visits. During follow up, because of limiting numbers of study visits, expression or nonexpression of the FP at a single study visit was used to define frailty and nonfrailty, respectively.

Subjects for the present study were selected from participants at the Baltimore-Washington, DC MACS site, who had: a) known frailty status by the above criteria; b) adequate availability of stored PBMCs; and c) for HIV+ men, undetectable HIV viral load (less than 50 copies/mL by the Roche ultrasensitive assay, Roche Diagnostics, Nutley, NJ) with cART. The selected population included 42 men (20 HIV-, 22 HIV+) and has been described in detail elsewhere [16]. Twenty-two men had been tested for CMV antibodies, and all were CMV-seropositive.

### Assessment of T cell responses to CMV peptide pools

T cell responses to peptide pools spanning 19 CMV ORFs (Supplementary Table 1), which consisted of 26 pools of overlapping 15-mer peptides (generously provided by Dr. Louis Picker) [14], were tested at baseline as described [15]. Briefly, cryopreserved PBMCs were thawed, rested overnight at 37 °C, and stimulated with peptide pools (0.2 μg/mL each) for 6 h in the presence of co-stimulatory anti-CD28 and anti-CD49d (1 μg/mL each, BD Biosciences, San Jose, CA). Brefeldin A (10 μg/mL, Sigma Aldrich) was added after 1 h of stimulation. Cultures containing staphylococcal enterotoxin B (SEB) and no peptides served as positive and negative controls, respectively.

Following stimulation and overnight storage at 4 °C, cytokine-producing T cells were identified by flow cytometry as described [15]. Briefly, cells were stained with LIVE/DEAD Fixable Aqua Dead Cell (Invitrogen, Eugene, OR), fixed and permeabilized using the Fixation/Permeabilization solution kit (Cytofix/Cytoperm™, BD Biosciences), and stained for 45 min with the following monoclonal antibodies: anti-IFN-γ–fluorescein isothiocyanate (FITC), anti-CD69–phycoerythrin (PE), anti-CD8–peridinin chlorophyll protein-Cyanine 5.5 (PerCP-Cy5.5), anti-CD3–allophycocyanin (APC), anti-IL-2–PE-Cy7, anti-TNF-α–Alexa Fluor 700, and anti-CD4–V450 (all antibodies, including isotype controls for anti-IFN-γ, anti-CD69, anti-IL-2, and anti-TNF-α, from BD Biosciences). Data were acquired on a LSRII cytometer (BD Biosciences) and analyzed using FlowJo software (FlowJo, Ashland, OR). Gates for cytokine- and CD69-staining were based on fluorescence of SEB-stimulated cells stained with the above antibodies to CD3, CD4, and CD8 and viability dye, along with isotype controls for antibodies to CD69, IFN-γ, TNF-α, and IL-2. Gates were further adjusted if background staining for IFN-γ, TNF-α, and/or IL-2 was higher in unstimulated cells than in SEB-stimulated cells stained with isotype control antibodies for these cytokines. The gating strategy is shown in Supplementary Fig. 1. Percentages of cytokine-producing T cells in negative controls were subtracted from those in samples stimulated with peptide pools to account for non-specific staining. Percentages ≥ 0.05% after subtraction were considered positive. To calculate the total percentage of a given cytokine-producing T cell subset for a donor, percentages of that subset among CD4 or CD8 T cells were summed across results obtained from cultures stimulated with all 26 peptide pools. Results were expressed as percentages of CD4 or CD8 T cells.

For some analyses, the 19 CMV ORFs studied were classified into 5 categories based on the function of their encoded products: glycoproteins, matrix, capsid, regulatory, and unknown (Supplementary Fig. 2 a and b) [14, 31, 32].

### Statistical analysis

**Fig. 1:**
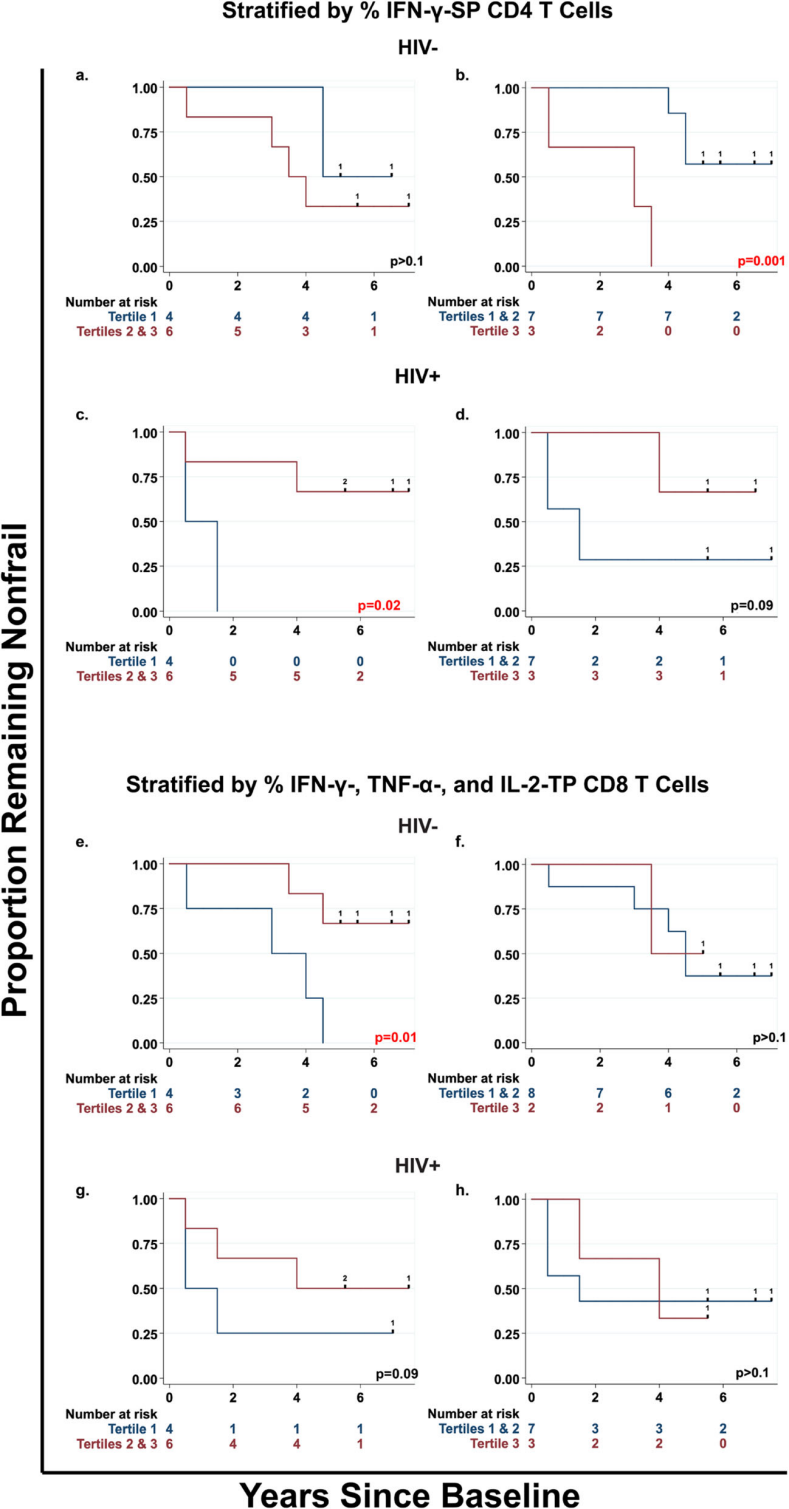
Effect of subsets of CMV-responsive T cells that significantly predicted onset of frailty. Shown are Kaplan-Meier survival curves (unadjusted) depicting onset of frailty among HIV- and HIV+ men, stratified by tertiles (1 = lowest, 3 = highest) of percentages of IFN-γ-SP CD4 T cells (**a-d**) and of IFN-γ-, TNF-α-, and IL-2- (triple)-producing CD8 T cells(**e-h**). The left column compares men in the lowest tertile of these percentages to those in the upper two tertiles; the right column compares men in the lower two tertiles to those in the top tertile among HIV- (**a, b and e, f**) and HIV+ (**c, d and g, h**) men. The number at risk indicates the number of people who were at risk of onset of frailty at the beginning of each time point. The *P*-values shown were obtained from the exact log-rank test; values in red indicate statistical significance. Hash marks and the numbers on survival curves indicate censoring times and the numbers of individuals censored at a given time point, respectively

**Fig. 2:**
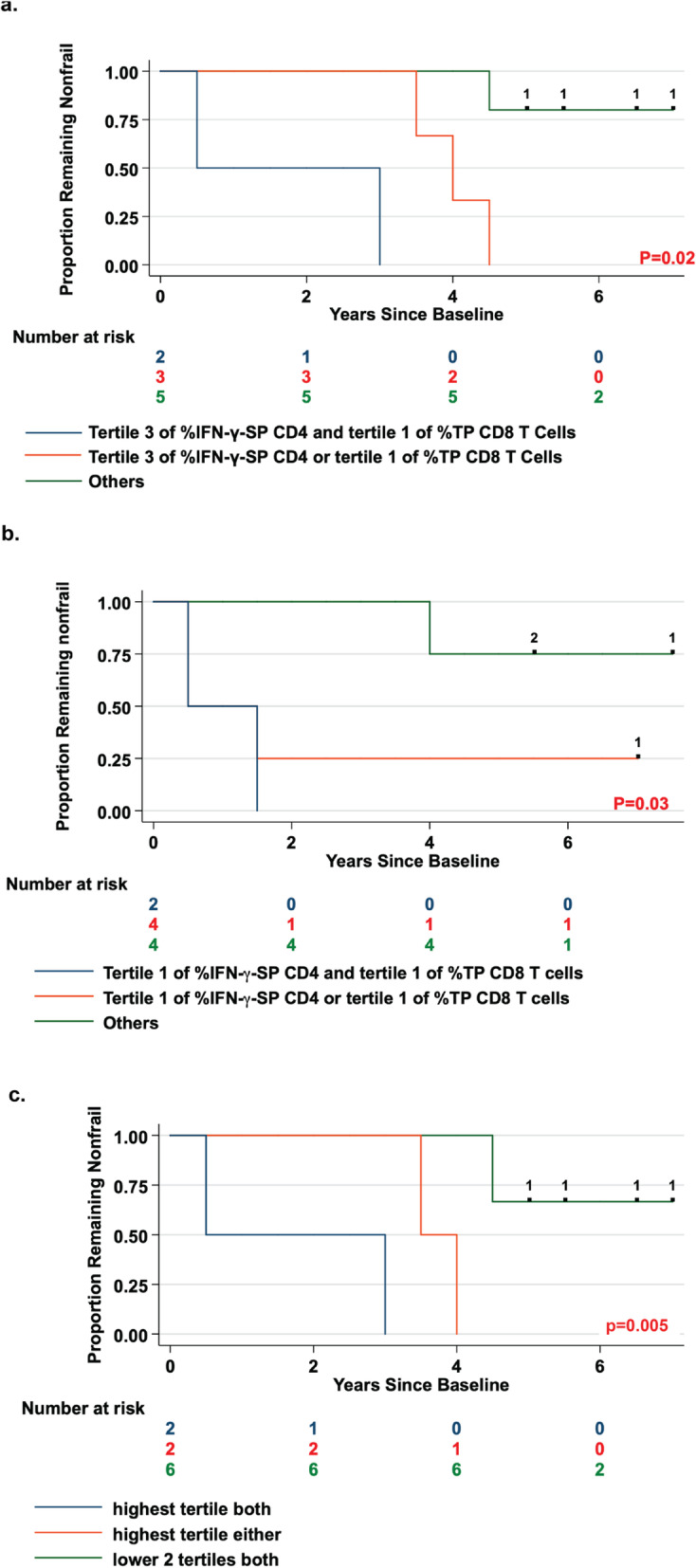
Prediction of frailty in HIV- nonfrail men by combinations of two subsets of CMV-specific cytokine-producing T cells. Kaplan-Meier survival curves (unadjusted) show the onset of frailty stratified by tertiles (1 = lowest, 3 = highest) of percentages of IFN-γ-SP CD4 T cells and TP CD8 T cells among HIV- nonfrail men (**a**) and among HIV+ nonfrail men (**b**), and by tertiles of IFN-γ-SP CD4 T cells and total IL-2-producing CD4 T cells among HIV- nonfrail men (**c**). In (**a**), HIV- nonfrail men were divided into three groups: those in both the highest tertile of IFN-γ-SP CD4 T cells and the lowest tertile of triple cytokine-producing CD8 T cells (blue line); those in either the highest tertile of IFN-γ-SP CD4 T cells or the lowest tertile of TP CD8 T cells, but not both (red line); and all other HIV- nonfrail men (green line). In (**b**), HIV+ nonfrail men were divided into those in the lowest tertile of both cell subsets (blue line), those in the lowest tertiles of either cell subset, but not. Both (red line), and those in the higher 2 tertiles of both cell subsets (green line). In (**c**), HIV- nonfrail men were divided into those in the highest tertile of both IFN-γ-SP CD4 T cells and total IL-2-producing CD4 T cells (blue line), those in the highest tertiles of either cell subset (red line), and those in the lower 2 tertiles of both cell subsets (green line). *P*-values shown in a-c were obtained by Fisher’s exact test. Numbers at risk, hash markers, and numbers on the survival curves are as described in legend of Fig. 1

**Fig. 3:**
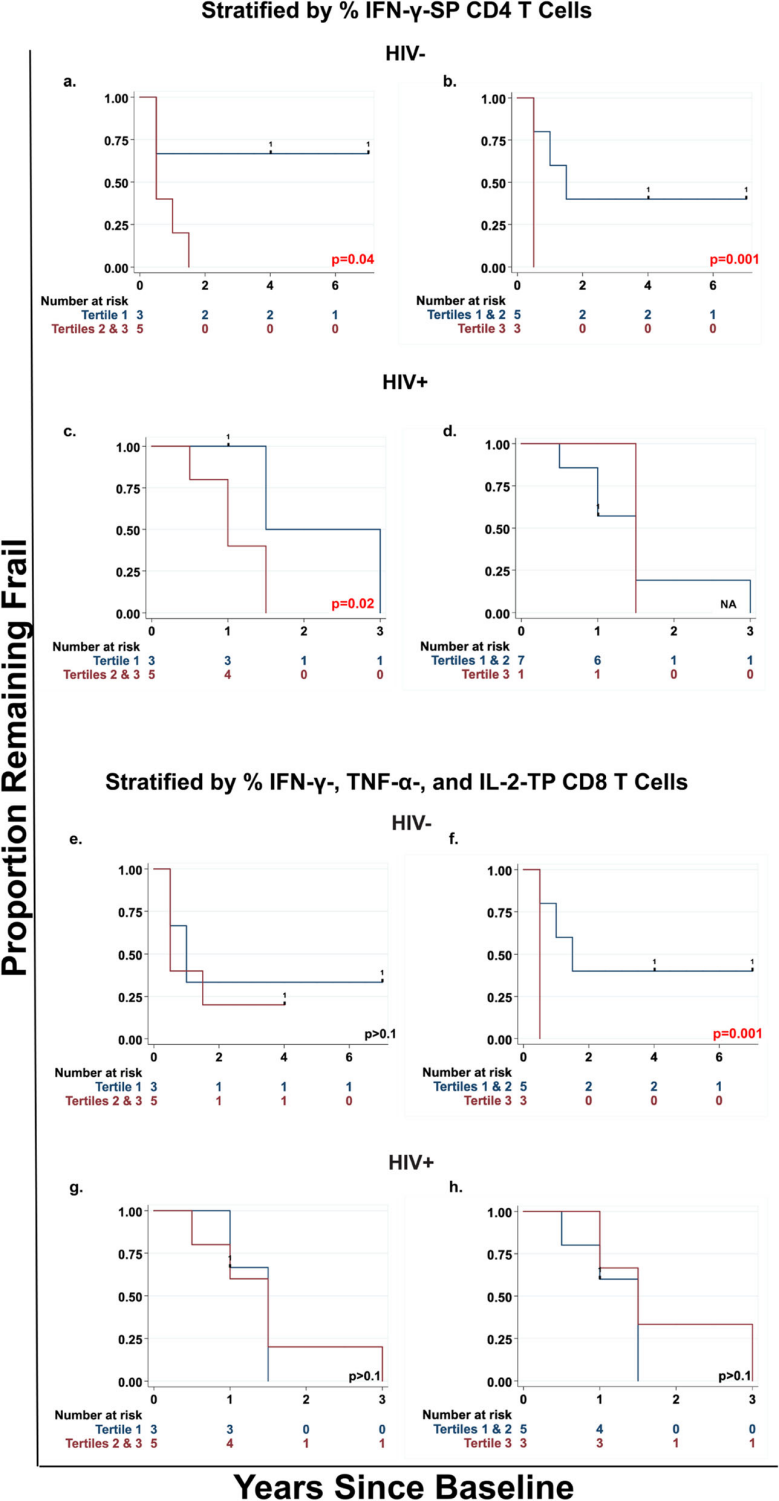
Prediction of remaining frail in HIV- frail men by IFN-γ-SP CD4 and TP CD8 subsets of CMV-responsive T cells. Kaplan-Meier survival curves (unadjusted) show the proportion remaining frail among HIV- and HIV+ men, stratified by tertiles (1 = lowest, 3 = highest) of percentages of IFN-γ-SP CD4 T cells (**a-d**) and of TP CD8 T cells (**e-h**). The left column shows comparison of men in the lowest tertile of these percentages versus those in the upper two tertiles among HIV- (**a and e**) and HIV+ (**c and g**) men. The right column shows comparison of men in the top tertile versus those in the lower two tertiles among HIV- (**b and f**) and HIV+ (**d and h**) men. Numbers at risk, *p* values, hash markers, and numbers on the survival curves are as described previously in legend of Fig. 1. Statistical testing could not be performed for d because only one man was in the top tertile of percentages of IFN-γ-SP CD4 T cells

**Fig. 4:**
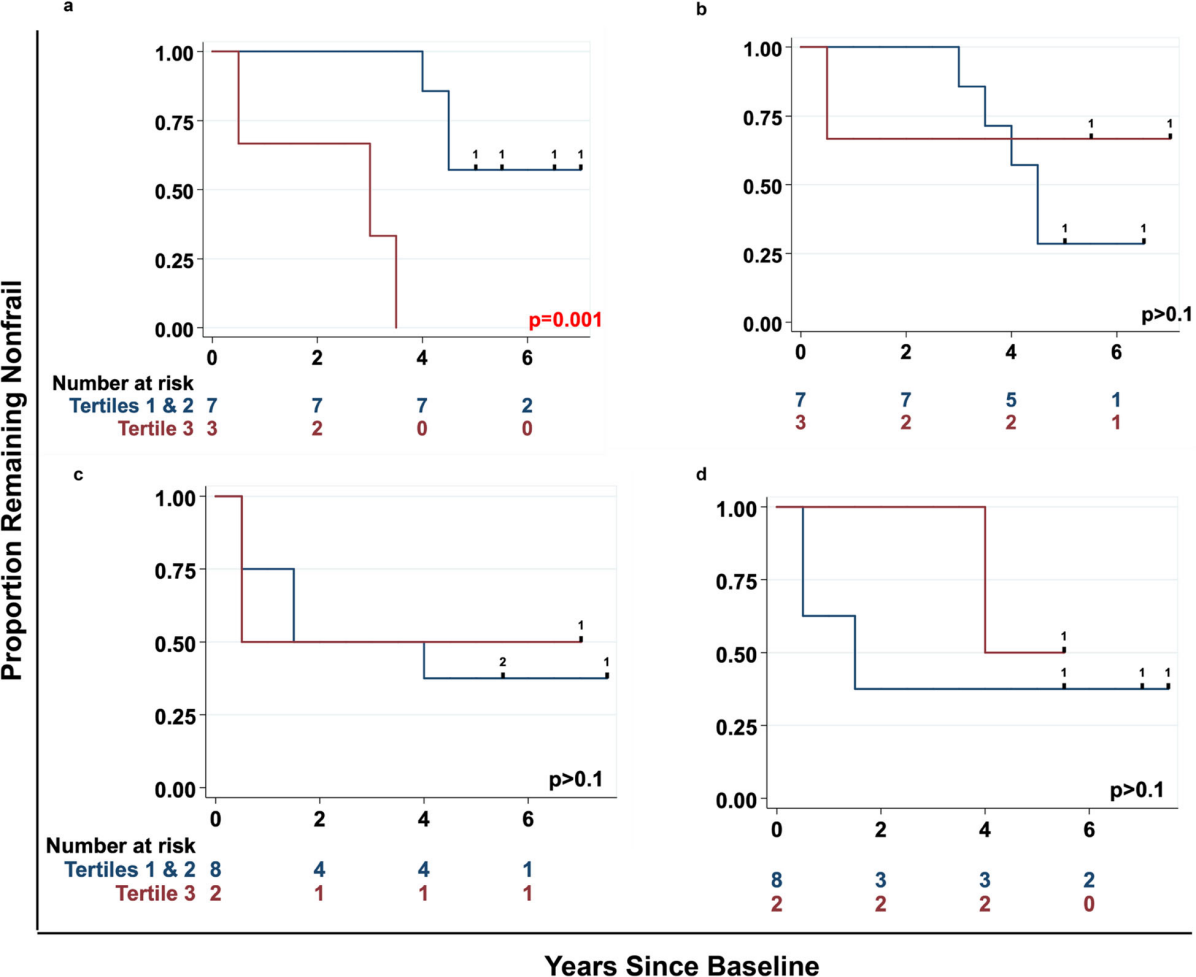
Prediction of onset of frailty in HIV- and HIV+ nonfrail men by tertiles (1 = lowest, 3 = highest) of percentages of IFN-γ-SP CD4 T cells responding to overlapping peptides spanning the CMV UL55 and US3 open reading frames. Kaplan-Meier survival curves (unadjusted) showing the proportion remaining nonfrail among nonfrail men, stratified by percentages of UL55-specific IFN-γ-SP CD4 T cells (a for HIV- and c for HIV+ nonfrail men) and US3-specific IFN-γ-SP CD4 T cells (b for HIV- and d for HIV+ nonfrail men), comparing the highest tertile vs the lower 2 tertiles. Numbers at risk, *p* values, hash markers, and numbers on the survival curves are as described in the legend to Fig. 1

Study subjects were divided into 4 groups at baseline: HIV- nonfrail, HIV- frail, HIV+ nonfrail, and HIV+ frail. The Kruskal–Wallis Test (for 3 or more groups) and Mann–Whitney U Test (for 2 groups) were used to assess significance of differences among groups for continuous variables. In each group, times from baseline to first occurrence of frailty (for men nonfrail at baseline) or to first occurrence of nonfrailty (for men frail at baseline) were compared between men with high or low percentages and absolute counts of cytokine-producing T cells (defined by tertiles), using Kaplan-Meier estimators and the exact log-rank test with multiple imputations of survival and censoring times because of the small sample size [33] (Figs. 1, 3, and 4). The cut-off values for tertiles are shown in Supplementary Table 2. For testing differences in time to onset of frailty among three groups, we compared the proportions who were frail at the last year before any censoring had occurred (e.g., year 4.5 for occurrence of frailty) using Fisher’s exact test (Fig. 2). For men who were frail at baseline, differences in proportions of follow-up visits at which the frailty phenotype was expressed were compared between tertiles of cytokine-producing cells using the Mann–Whitney U Test (Table 1). To make the best use of the relatively small sample, we analyzed the tertiles in two ways: highest tertile vs lower 2 tertiles, and lowest tertile vs higher 2 tertiles. Correlations between percentages of cytokine-producing T cells and serum levels of inflammatory markers were explored using nonparametric Spearman’s correlation coefficients.

**Table 1:** Proportion of follow-up visits with manifestation of frailty among HIV- and HIV+ men who were frail at baseline, stratified by tertiles of IFN-γ-SP CD4 and TP CD8 subsets of CMV-responsive T cells

	HIV- frail (*n* = 8)			HIV+ frail (*n* = 8)		
	Proportions of frail visits (%)		*P* value	Proportions of frail visits (%)		*P* value
	Highest tertile	Lower 2 tertiles		Higher 2 tertiles	Lowest tertile	
IFN-γ-SP CD4	50 (31.4)^a^	83.3 (35.7)	0.1	28.6 (43.3)	93.3 (62.5)	0.1
TP CD8	37.5 (21.4)	83.3 (35.7)	0.02	60 (35.7)	37.5 (51.4)	0.88

Note. These proportions were calculated among men who had more than 1 follow-up visits after baseline

^a^Median (IQR)

Analyses were performed using Stata version 14.0 (StataCorp, College Station, TX) and R version 3.6.3. A *p* value of <.05 was considered statistically significant.

## Results

### Study population

Demographic and clinical characteristics of the study subjects were reported previously [16] and are summarized in Supplementary Table 3. HIV- men were slightly older than HIV+ men (median ages = 63 yr vs 58 yr, respectively; *p* < .05), but other characteristics, including educational level, ethnicity, smoking status, depressive symptoms, chronic diseases (liver, kidney, diabetes, hypertension), and chronic infections (prevalence of hepatitis C virus [HCV] and titer of anti-CMV IgG antibody), did not differ significantly by HIV infection status. By design, all of the HIV+ men had undetectable HIV viral loads (< 50 copies/mL).

Frailty status of men was assessed semiannually for a median of 6.5 years (IQR: 2, ranges from 0 to 7.5 years) after baseline assessment of CMV-specific T cell responses. Length of follow-up was similar among the four groups of participants (data not shown) .

### Polyfunctional T cell responses to CMV

With three cytokines assessed (IFN-γ, TNF-α, and IL-2), 7 subpopulations of cytokine-producing CMV-responsive T cells were theoretically possible among CD4 and CD8 T cells. Of these, three subpopulations accounted for nearly all of the responding cells across all subjects (medians of 95.9% of total responding CD4 T cells (IQR: 27.8%) and 99.3% of total responding CD8 T cells (IQR: 6.2%), Supplementary Fig. 2): IFN-γ- and TNF-⍺-double-producers (median (IQR): 50.6 (34.3)% and 57.7 (21.3) % of total responding CD4 and CD8 cells, respectively), IFN-γ-single-producers (14.8 (24.0)% and 34.2 (23.0)%), and IFN-γ-, TNF-⍺-, and IL-2-triple-producers (11.4 (21.5)% and 4.5 (6.0) %). Given this strong predominance, further analyses were restricted to these three subsets of CD4 and CD8 T cells, for a total of six subsets analyzed. The percentages of these subsets for all CMV ORFs are shown in Supplementary Fig. 3.

### Men who were nonfrail at baseline: CMV-specific cytokine responses and onset of frailty

Baseline levels of two of the six predominant subsets of CMV-responsive T cells significantly predicted faster onset of frailty in both HIV- and HIV+ men: IFN-γ-single-producing (SP) CD4 T cells and triple-producing (TP) CD8 T cells.

In HIV- men, faster onset of frailty was predicted by higher percentages of IFN-γ-SP CD4 T cells (Fig. 1a and b), and lower percentages of TP CD8 T cells (Fig. 1e and f). Men who exhibited both high percentages of IFN-γ-SP CD4 T cells and low percentages of TP CD8 T cells had faster onset of frailty than in those who exhibited one, or neither, of these findings, and the difference was significant (Fig. 2a, *p* = 0.02). Men who exhibited both or either of these findings comprised 5 of the 6 nonfrail HIV- men who progressed to frailty by the end of year 4.5 (Fig. 2a). The significance of these predictions depended on the tertiles being compared, likely because of variations in numbers of participants in the different comparison groups. However, the directions of the predictions were the same for all tertile comparisons.

In HIV+ men, lower, rather than higher, percentages of IFN-γ-SP CD4 T cells predicted faster onset of frailty (Fig. 1c and d). Lower percentages of TP CD8 T cells were again suggestive of faster onset of frailty, but not significantly so (Fig. 1g and h). As in HIV- men, HIV+ men who had lower percentages of either subset comprised 5 of the 6 HIV+ nonfrail men who progressed to frailty by the end of year 4 (Fig. 2b).

We repeated these analyses using the absolute counts of IFN-γ-SP CD4 T cells and TP CD8 T cells (cells/μL) rather than the percentages of these cells. The predictions obtained were essentially unchanged, in both HIV- and HIV+ nonfrail men (Supplementary Fig. 4). This reflects the high concordance of categorization of the men into tertiles using percentages and absolute counts (Supplementary Table 4).

Because a previous analysis of the subjects in this study found that higher percentages of total IL-2-producing CMV-specific CD4 T cells predicted faster onset of frailty in HIV- nonfrail men [16], we examined if any of the polyfunctional CD4 T cell subsets producing IL-2 (i.e., IL-2-SP, triple-producers, and IFN-γ- and IL-2-double producers) could predict onset of frailty. None of these subsets was predictive (data not shown). Accordingly, we examined whether IFN-γ-SP and total IL-2-producing CMV-specific CD4 T cells together could predict onset of frailty in HIV- nonfrail men. As shown in Fig. 2c, onset of frailty among men in the highest tertile of one or both of these CD4 T cell responses was faster than in men not in the top tertile for either response, and the difference among these three groups was significant (*p* = 0.005).

### Men who were frail at baseline: CMV-specific T cell cytokine responses and maintenance of frailty

Maintenance of frailty was evaluated through two variables: time to the first nonfrail visit, and the proportion of follow-up visits at which the donor was frail.

In HIV- men, the two CMV-induced T cell subsets that predicted onset of frailty also predicted maintenance of frailty. Specifically, both lower percentages of IFN-γ-SP CD4 T cells and lower percentages of TP CD8 T cells significantly predicted longer time to the first nonfrail visit (Fig. 3a and b, and Fig. 3e and f, respectively) and a higher median proportion of follow-up visits manifesting frailty (Table 1).

In HIV+ men, lower percentages of IFN-γ-SP CD4 T cells again predicted both longer maintenance of frailty (*p* = .02; Fig. 3c) and higher median proportions of follow-up visits manifesting frailty (Table 1). However, TP CD8 T cells did not predict maintenance of frailty in any analysis (Fig. 3g and h, and Table 1).

As with onset of frailty, the analysis of absolute counts of IFN-γ-SP CD4 T cells and TP CD8 T cells predicted maintenance of frailty similarly to the percentages of these cells, except that prediction by TP CD8 in HIV- men was no longer significant (Supplementary Fig. 5).

The significant relationships found are summarized in Supplementary Table 5. In HIV- men lower percentages of TP CD8 T cells were associated with both faster onset and longer maintenance of frailty, while higher percentages of IFN-γ-SP CD4 T cells predicted faster onset, but shorter maintenance, of frailty. In HIV+ men, in contrast, the direction of the association between IFN-γ-SP CD4 T cells and both maintenance and onset of frailty was negative.

### Differential antigenic specificity of CMV-induced T cell responses in HIV- and HIV+ nonfrail men

It was notable that higher percentages of IFN-γ-SP CD4 T cells predicted faster onset of frailty in HIV- nonfrail men but slower onset in HIV+ nonfrail men. To explore this finding, we asked whether the CMV ORFs that elicited this response differed by HIV status. Indeed, ORFs encoding CMV glycoproteins elicited this response to a greater degree in HIV- than in HIV+ nonfrail men (median (IQR) percentages among CD4 T cells = 0.33 (0.42) and 0.05 (0.26), respectively; *p* = .05; Supplementary Fig. 6a). Further, the response to these ORFs also represented a significantly greater proportion of the total IFN-γ-SP CD4 T cell response in HIV- than in HIV+ nonfrail men (median (IQR) percentages = 57.1 (35.5) and 17.6 (29.9), respectively; *p* = .02; Supplementary Fig. 6b). The reverse was true for ORFs encoding matrix proteins (median (IQR) percentages = 17.3 (41.9) and 48.4 (46.1), respectively; *p* = .04, Supplementary Fig. 5b). These responses did not differ by HIV status in frail men (data not shown).

Among the 19 CMV ORFs studied, UL55 (gB), US3, and US29 code for the CMV glycoprotein. UL55 elicited much greater responses than US3 and US29 (Supplementary Table 6), and UL55-specific responses were significantly more frequent and larger in HIV- than in HIV+ nonfrail men (Supplementary Table 6). Moreover, a higher percentage of UL55-specific IFN-γ-SP CD4 T cells significantly predicted faster onset of frailty in HIV-nonfrail men (Fig. 4a; *p* = .001) but not HIV+ nonfrail men (Fig. 4c). CD4 IFN-γ-only responses to US3 and US29 were less frequent, lower, and not different between HIV- and HIV+ nonfrail men, and did not predict onset of frailty in either group (Fig. 4b for HIV- nonfrail men, and Fig. 4d for HIV+ nonfrail men).

### Differential correlations between CMV-induced T cell responses and serum inflammatory markers in HIV- and HIV+ nonfrail men

We asked if percentages of cytokine-producing T cells elicited by CMV peptide pools, which we previously found to be significantly correlated with serum levels of inflammatory markers depending on HIV and frailty status [16], were correlated with serum inflammatory markers differently in HIV- and HIV+ nonfrail men. Of the 16 serum markers (cytokines, chemokines, and CRP) for which data were available, the main finding was that the correlation between percentages of IFN-γ-SP CD4 T cells and serum levels of IFN-γ was much stronger in HIV+ nonfrail men (*r* = 0.83, *p* = .001) than in HIV- nonfrail men (*r* = 0.28, *p* > .1; p for difference in correlation coefficients = .08 by Fisher’s z transformation, Supplementary Fig. 7a). Also, correlations of these cells with serum CRP and IL-10 were in different directions in HIV- and HIV+ nonfrail men (for CRP: *r* = 0.40 and − 0.09, respectively; p for difference > .1; for IL-10: *r* = − 0.44 and 0.58, respectively; p for difference = 0.03, Supplementary Fig. 7, b and c). In addition, percentages of TP CD8 T cells were negatively correlated with CRP, strongly in HIV- nonfrail men (*r* = − 0.64, *p* = 0.04), but less so in HIV+ nonfrail men (*r* = − 0.22, *p* > 0.1, Supplementary Fig. 7d).

Finally, we explored if the serum levels of the above three inflammatory markers could predict onset and/or maintenance of frailty, considering their correlations with CMV-specific cytokine-producing T cells. The results are summarized in Supplementary Table 7. Among nonfrail men, higher levels of CRP predicted faster onset of frailty only in HIV- men (Supplementary Fig. 8), while lower levels of IFN-γ and IL-10 made this prediction only in HIV+ men (Supplementary Figs. 9 and 10). Among frail men, higher levels of CRP and lower levels of IL-10 predicted greater maintenance of frailty only in HIV- men (Supplementary Fig. 11 and Supplementary Table 8). Notably, percentages of IFN-γ-SP CD4 T cells were correlated negatively with levels of CRP (*r* = − 0.58, *p* = 0.09) and positively with levels of IL-10 (*r* = 0.5, *p* > 0.1) in HIV- frail men.

Besides the above three inflammatory markers, we found five other markers (Eotaxin-3, IL-8, MIP-1⍺, TARC, and TNF-⍺) that also predicted onset or maintenance of frailty. These predictions were also restricted to either HIV- or HIV+ men, as for CRP, IFN-γ and IL-10; and these predictions were not related to their correlations with either CMV-specific T cell subset (data not shown).

## Discussion

This study investigated the relationship between polyfunctional CMV-specific T cell responses and onset and maintenance of frailty in HIV- and virologically suppressed HIV+ men. This study extends our previous analysis of the same population [16] by evaluating: 1) the polyfunctional T cell cytokine response to CMV antigens, and 2) its relationship with maintenance as well as onset of frailty.

We found that six out of the 14 possible functional subsets of CMV-responsive CD4 and CD8 T cells accounted for almost all CMV-responsive cells. This predominance was not substantially affected by HIV or frailty status. Of these six subsets, percentages of two, CD4 T cells producing only IFN-γ (IFN-γ-SP CD4 T cells) and CD8 T cells producing IFN-γ, TNF-α, and IL-2 (TP CD8 T cells), significantly predicted onset and maintenance of frailty in both the HIV- and the HIV+ men.

In HIV- men who were nonfrail at baseline, higher percentages of IFN-γ-SP CD4 T cells and lower percentages of TP CD8 T cells predicted faster onset of frailty. Further, a) men who had both high percentages of IFN-γ-SP CD4 T cells and low percentages of TP CD8 T cells progressed to frailty faster than other men; b) such men comprised most of those who progressed to frailty; and c) serum levels of CRP, which have been associated with CMV infection and frailty [8, 34], were correlated positively with percentages of IFN-γ-SP CD4 T cells and negatively with percentages of TP CD8 T cells. Since polyfunctional T cells have been shown to exert stronger anti-CMV effects than single cytokine-producing T cells [18, 24, 35], these data suggest that insufficient control of CMV infection may contribute to the development of frailty, and that the T cell response to CMV may contribute to the well-known association of inflammation with frailty.

This interpretation is strengthened by the findings that a large percentage of IFN-γ-SP CD4 T cells were elicited by peptides encoded by CMV UL55 (gB), and higher percentages of UL55-specific IFN-γ-SP CD4 T cells predicted faster onset of frailty. UL55-specific CD4 T cells have been reported to be more cytolytic than CD4 T cells specific for other CMV proteins (i.e., UL83 (pp65), UL86, UL115 (gL), and UL75 (gH)) [20, 21, 26], and to express high levels of the vascular endothelium homing receptor CX3CR1 [26, 36]. Considering the importance of TP CD8 T cells in controlling CMV reactivation [18], low levels of these cells may lead to more CMV reactivation, which could in turn trigger higher levels of UL55-specific IFN-γ-SP CD4 T cells. Thus, one could postulate that suboptimal control of CMV reinfection or reactivation could lead to increased inflammation and vascular damage [26, 36, 37], both of which can predispose to frailty [8, 10]. The strong response of IFN-γ-SP CD4 T cells to UL55 may explain our finding that in HIV- men percentages of IFN-γ-SP CD4 T cells were correlated positively with serum levels of CRP and negatively with serum levels of IL-10. Since IL-2 can increase proliferation and survival of UL55-specific cytolytic CD4 T cells [38], this could explain why high levels of IL-2-producing CD4 T cells and IFN-γ-SP CD4 T cells together predicted progression to frailty more strongly than either subset individually.

Unexpectedly, in HIV+ men it was lower, rather higher, percentages of IFN-γ-SP CD4 T cells that predicted faster onset of frailty. The reason for this difference is unclear. However, the differences between HIV- and HIV+ nonfrail men in antigenic specificity of these cells and their correlations with serum inflammatory markers may offer clues. In contrast to HIV- men, in HIV+ men most IFN-γ-SP CD4 T cells were not specific to UL55, and percentages of these cells specific to UL55 did not predict onset of frailty. Also, the percentages of these cells were not correlated with CRP. However, these percentages were positively correlated with the serum levels of the anti-inflammatory cytokine IL-10 and of IFN-γ. IL-10 was protective against frailty in a mouse model with IL-10 knockout [39], and circulating IFN-γ inhibits CMV replication [40]. Thus, in HIV+ men lower levels of these cells could predispose to induction of frailty-associated inflammation by CMV. Other differences between HIV+ and HIV- men, including numerical and functional differences in T cell subsets, and/or potential sites and nature of CMV antigen production, could also have contributed to the difference in CMV responses.

Lower percentages of TP CD8 T cells were associated with faster onset of frailty in both HIV- and HIV+ men, although this was statistically significant only for the former. This weaker effect in HIV+ men may indicate that the protective effect of TP CD8 cells is disrupted by chronic HIV infection, or it could reflect the small sample size of the study. Nevertheless, the majority (5 out of 6) of HIV+ men with progression to frailty had low percentages of both IFN-γ-SP CD4 T cells and TP CD8 T cells, suggesting a combined effect of these two cell subsets on onset of frailty in HIV+ men as in HIV- men. In HIV+ men, however, both of these cell subsets may protect against CMV reactivation and prevent CMV-induced inflammation. CMV-specific CD4 T cells have been shown to be required for dendritic cell-mediated activation of cognate CD8 T cell responses, partly through production of IFN-γ by CD4 T cells [41]. Therefore, the combined effect of both T cell subsets may suggest the importance of CD4 help in promoting and sustaining effective anti-viral CD8 T cell responses. This interpretation may also explain the weaker effect of TP CD8 T cells we observed in HIV+ men, as it is possible that HIV provirus in CMV-specific CD4 T cells could become lytic after these cells are activated by CMV antigens [42].

For both HIV- and HIV+ men who were frail at baseline, lower levels of IFN-γ-SP CD4 T cells predicted greater maintenance of frailty, while lower levels of TP CD8 T cells were predictive only in HIV- men. These results provide additional evidence suggesting the importance of these two T cell subsets in the pathogenesis of frailty. They also suggest that chronic HIV infection may disrupt the protective effect of TP CD8 T cells, and that controlling CMV reactivation may be key for reversal of frailty, regardless of HIV infection.

We found that the serum inflammatory markers CRP, IL-10, and IFN-γ, which were correlated with the percentages of the two predictive CMV-specific T cell subsets, also predicted onset and/or maintenance of frailty. The predictions were restricted to either HIV- or HIV+ men, and directions of the predictions were consistent with the CMV-specific T cell subsets with which they were correlated. The directions of the predictions by CRP and IL-10 were also consistent with previous reports [43, 44].

Overall, the results of this study support the hypothesis that CMV-induced inflammation is an important contributory mechanism for the observed association between CMV-specific T cells and frailty. However, no causation can be inferred from the cross-sectional correlations between inflammatory markers and CMV-specific T cells. Moreover, an additional hypothesis that is consistent with the data is that inflammation activates CMV replication which in turn elicits the T cell responses observed in this study.

This study had several limitations. The sample size was small, and the findings, though statistically significant, should be confirmed in larger studies. We did not adjust our statistical analyses for multiple comparisons, because this increases the likelihood of false-negative results with a small sample size [45]. Although not adjusting can lead to false-positive results, the likelihood of this is reduced by the consistency of the directions of associations between functional T cell subsets and onset or maintenance of frailty across different tertile comparisons, and of differences in the proportions of follow-up visits at which the participants were frail. The study is also limited by unavailability of data on clinical conditions that could have affected the development of frailty, and by the lack of women in the study population. Another limitation is that while half of the study population was known be CMV-seropositive, the serostatus of the other half was not known. However, since all had positive CMV-specific T cell responses and nearly all HIV-infected people are CMV-seropositive [46], it is highly likely that all the men studied were CMV-seropositive. This study also had some notable strengths, including: 1) the prospective study design and long follow-up period, which reduce bias and permit temporal inference; 2) exploration of both onset and maintenance of frailty since few studies have investigated the maintenance of frailty; 3) a large panel of peptide pools spanning 19 CMV ORFs was used to assess CMV-specific T cells, permitting a much more comprehensive detection of these cells than in most studies, which have assessed only responses to UL83 and/or UL123, which usually represent a minority of anti-CMV T cell responses [14, 15, 47].

## Conclusions

The findings in this study suggest a temporal relationship between the functional T cell response to CMV and both onset and maintenance of frailty, which differs by HIV status of the host. Expansion of CMV-specific T cells has been associated with aging and immunosenescence [48]. Here we showed that lower levels of two functional T cell subsets predicted faster onset and, at least in some cases, greater maintenance of frailty, suggesting that manipulation of these functional subsets could prevent or inhibit CMV reactivation and reduce chronic inflammation induced by CMV reactivation. Further work is needed to confirm these findings and to define how different cytokine-producing subsets of CMV-responsive cells are generated and maintained in HIV- and HIV+ people, and how this relates to CMV latency, reactivation, and antigenic expression. In addition, these CMV-specific functional subsets have the potential to serve as biomarkers for predicting frailty in both HIV- and HIV+ people, although this too must be validated in larger studies.

## Supplementary Information

**Additional file 1: Supplementary Figure 1.** Gating strategy for identifying single-, double-, and triple-cytokine-producing CD8 T cells. After identifying viable CD8 T cells via forward scatter/side scatter and expression of Aqua LIVE/DEAD dye, CD3, and CD8, production of IFN-γ, and/or TNF-α, and/or IL-2 was measured in CD69+ CD8 T cells as shown. The percentage of a given cytokine-producing CD8 T cell subset among CD8 T cells was subsequently calculated as the product of the percentage of the first gating quadrant and the proportion of the second gating quadrant used to define the subset. For example, calculation of the percentages of TNF-⍺-single-producing and of IFN-γ- and TNF-α-double-producing CD8 T cells is shown. The same gating strategy was applied to CD4 T cells. **Supplementary Figure 2.** Percentages of the three most common cytokine-producing phenotypes, i.e., IFN-γ-single-producing (SP), IFN-γ- and TNF-α-double-producing (DP), and IFN-γ-, TNF-α, and IL-2-triple-producing (TP), and the sums of the percentages of these three phenotypes, among total CMV-specific CD4 (black) and CD8 (red) T cells, stratified by HIV status and frailty status. Each circle represents one donor. The median, IQR, and range for each donor group are indicated by the boxplots. **Supplementary Figure 3.** Percentages of IFN-γ-SP, IFN-γ- and TNF-α-DP, and IFN-γ-, TNF-α-, and IL-2-TP generated in response to each of the 19 CMV ORFs, stratified by HIV status and frailty status, among CD4 (a) and CD8 (b) T cells. Each circle represents one donor, and the median, IQR, and range for each ORF are indicated by the boxplots. The percentages are log_10_-transformed to enhance visualization. The red dashed lines indicate the threshold of detection of CMV-responsive cells (0.05%). **Supplementary Figure 4.** Prediction of remaining nonfrail in HIV- frail men by absolute counts of IFN-γ-SP CD4 and TP CD8 subsets of CMV-responsive T cells. Kaplan-Meier survival curves (unadjusted) show the proportion remaining nonfrail among HIV- and HIV+ men, stratified by tertiles of absolute counts of IFN-γ-SP CD4 T cells (a-d) and of TP CD8 T cells (e-h). The left column shows comparisons of men in the lowest tertile of these numbers versus those in the upper two tertiles. The right column shows comparisons of men in the top tertile versus those in the lower two tertiles. Numbers at risk, *p* values, hash markers, and numbers on the survival curves are as described in the legend of Fig. 1. **Supplementary Figure 5.** Prediction of remaining frail in HIV- frail men by absolute counts of IFN-γ-SP CD4 and TP CD8 subsets of CMV-responsive T cells. Kaplan-Meier survival curves (unadjusted) show the proportion remaining frail among HIV- and HIV+ men, stratified by tertiles of absolute counts of IFN-γ-SP CD4 T cells (a-d) and of TP CD8 T cells (e-h). The left column shows comparisons of men in the lowest tertile of these numbers versus those in the upper two tertiles. The right column shows comparisons of men in the top tertile versus those in the lower two tertiles. Numbers at risk, *p* values, hash markers, and numbers on the survival curves are as described in the legend of Fig. 1. The *p* value could not be determined for figure d because only one man was in the top tertile. **Supplementary Figure 6.** Responsiveness of IFN-γ-SP CD4 T cells to CMV ORFs. Magnitude of the IFN-γ-SP CD4 T cell response to CMV open reading frames (ORFs) that were elicited by specific functional categories of CMV ORFs, expressed a) as a percentage of CD4 T cells, and b) as a percentage of the total CD4 IFN-γ-SP response. Data are shown from HIV- nonfrail men (hollow circles) and HIV+ nonfrail men (filled circles). The 19 CMV ORFs tested were categorized based on the function of their encoded proteins: glycoproteins (UL55, US3, and US29), matrix (UL32, UL36, UL82, UL83, UL94, UL99, UL103, US24, and UL28), capsid (UL48 and UL86), regulatory (UL122, UL123, and US32), and unknown (UL151 and UL153). **Supplementary Figure 7.** Correlations between percentages of CMV-responsive T cells and serum concentrations of inflammatory markers. a-c) Correlations between percentages of CMV-specific IFN-γ-SP CD4 T cells (among total CD4 T cells) and serum levels of IFN-γ (a), CRP (b), and IL-10 (c) in HIV- and HIV+ nonfrail men. d) the correlation between percentages of IFN-γ-, TNF-⍺-, and IL-2-TP CD8 T cells (among total CD8 T cells) and serum levels of CRP. Each point represents one donor, and the line represents the least squared regression line. Data were log_10_-transformed, with undetectable values coded as − 2. Nonparametric Spearman’s correlation coefficients are shown. **Supplementary Figure 8.** Prediction of becoming frail by serum levels of CRP in HIV- and HIV+ men. Kaplan-Meier survival curves (unadjusted) show the proportion remaining nonfrail among HIV- and HIV+ men ((a-d), stratified by tertiles of serum levels of CRP (mg/mL). The left column compares people in the lowest tertile of these percentages versus those in the upper two tertiles among HIV- (a) and HIV+ (c) men. The right column compares people in the top tertile versus those in the lower two tertiles among HIV- (b) and HIV+ (d) men. Number at risk, *p* values, hash markers, and numbers on the survival curves are as described in legend of Fig. 1. **Supplementary Figure 9.** Prediction of becoming frail by serum levels of IFN-γ in HIV- and HIV+ men. Kaplan-Meier survival curves (unadjusted) show the proportion remaining nonfrail among HIV- (a, b) and HIV+ men (c, d), stratified by tertiles of serum levels of IFN-γ (pg/mL). The left column shows people in the lowest tertile of these percentages versus those in the upper two tertiles among HIV- (a) and HIV+ (c) men. The right column compares people in the top tertile versus those in the lower two tertiles among HIV- (b) and HIV+ (d) men. Number at risk, *p* values, hash markers, and numbers on the survival curves are as described in legend of Fig. 1. **Supplementary Figure 10.** Prediction of becoming frail by serum levels of IL-10 in HIV- and HIV+ men. Kaplan-Meier survival curves (unadjusted) show the proportion remaining nonfrail among HIV- and HIV+ men ((a-d), stratified by tertiles of serum levels of IL-10 (pg/mL). The left column shows people in the lowest tertile of these percentages versus those in the upper two tertiles among HIV- (a) and HIV+ (c) men. The right column compares people in the top tertile versus those in the lower two tertiles among HIV- (b) and HIV+ (d) men. Number at risk, *p* values, hash markers, and numbers on the survival curves are as described in legend of Fig. 1. **Supplementary Figure 11.** Prediction of remaining frail in HIV- men by serum levels of CRP and IL-10. Kaplan-Meier survival curves (unadjusted) show the proportion remaining frail among HIV- and HIV+ men, stratified by tertiles of percentages of CRP (a-d) and of IL-10 (e-h). The left column shows men in the lowest tertile of these percentages versus those in the upper two tertiles among HIV- (a and e) and HIV+ (c and g) men. The right column compares men in the top tertile versus those in the lower two tertiles among HIV- (b and f) and HIV+ (d and h) men. Number at risk, *p* values, hash markers, and numbers on the survival curves are as described in legend of Fig. 1. The *P* value could not be determined for figure b because only one man was in the top tertile of CRP. **Supplementary Table 1.** Human CMV open reading frames (ORFs) of the peptide pools tested in this study^a^. **Supplementary Table 2.** Cut-off values of percentages and absolute counts of IFN-γ-SP CD4 T Cells and IFN-γ-, TNF-α-, and IL-2-TP CD8 T cells for defining tertiles. **Supplementary Table 3.** Demographic Characteristics of the Men Studied (*N* = 42). **Supplementary Table 4.** Concordance of categorization by tertiles using percentages and numbers of IFN-γ-SP CD4 T Cells (a) and IFN-γ-,TNF-α-, and IL-2-TP CD8 T cells (b). **Supplementary Table 5.** Significant predictors of adverse frailty-related outcomes by polyfunctional T cell subsets. **Supplementary Table 6.** IFN-γ-only CD4 T cell responses to peptide pools spanning CMV UL55, US3, and US29 open reading frames, among HIV- and HIV+ nonfrail men. **Supplementary Table 7.** Summary of prediction of onset and maintenance of frailty by serum levels of inflammatory markers. **Supplementary Table 8.** Proportion of follow-up visits with manifestation of frailty among HIV- and HIV+ men who were frail at baseline, stratified by tertiles of serum levels of CRP, IL-10, and IFN-γ.

## Data Availability

The datasets used and/or analyzed during the current study are available from the corresponding author on reasonable request.
