# Supplementary material for: Longitudinal association of cytokine-producing CMV-specific T cells with frailty in HIV-infected and -uninfected men who have sex with men
Source: Immun Ageing. 2022 Mar 7;19:13. doi: 10.1186/s12979-022-00270-3 (PMC8900335; doi:10.1186/s12979-022-00270-3)
Supplement: Supplementary file 1 — Additional file 1: Supplementary Figure 1. Gating strategy for identifying single-, double-, and triple-cytokine-producing CD8 T cells. After identifying viable CD8 T cells via forward scatter/side scatter and expression of Aqua LIVE/DEAD dye, CD3, and CD8, production of IFN-γ, and/or TNF-α, and/or IL-2 was measured in CD69+ CD8 T cells as shown. The percentage of a given cytokine-producing CD8 T cell subset among CD8 T cells was subsequently calculated as the product of the percentage of the first gating quadrant and the proportion of the second gating quadrant used to define the subset. For example, calculation of the percentages of TNF-⍺-single-producing and of IFN-γ- and TNF-α-double-producing CD8 T cells is shown. The same gating strategy was applied to CD4 T cells. Supplementary Figure 2. Percentages of the three most common cytokine-producing phenotypes, i.e., IFN-γ-single-producing (SP), IFN-γ- and TNF-α-double-producing (DP), and IFN-γ-, TNF-α, and IL-2-triple-producing (TP), and the sums of the percentages of these three phenotypes, among total CMV-specific CD4 (black) and CD8 (red) T cells, stratified by HIV status and frailty status. Each circle represents one donor. The median, IQR, and range for each donor group are indicated by the boxplots. Supplementary Figure 3. Percentages of IFN-γ-SP, IFN-γ- and TNF-α-DP, and IFN-γ-, TNF-α-, and IL-2-TP generated in response to each of the 19 CMV ORFs, stratified by HIV status and frailty status, among CD4 (a) and CD8 (b) T cells. Each circle represents one donor, and the median, IQR, and range for each ORF are indicated by the boxplots. The percentages are log10-transformed to enhance visualization. The red dashed lines indicate the threshold of detection of CMV-responsive cells (0.05%). Supplementary Figure 4. Prediction of remaining nonfrail in HIV- frail men by absolute counts of IFN-γ-SP CD4 and TP CD8 subsets of CMV-responsive T cells. Kaplan-Meier survival curves (unadjusted) show the proportion remaining [file 12979_2022_270_MOESM1_ESM.docx]

Supplementary Material

Longitudinal association of cytokine-producing CMV-specific T cells with frailty in HIV-infected and -uninfected men who have sex with men

Weiying Zhang, PhD^1^, Huifen Li, PhD^2^, Jay H. Bream, PhD^1, 3^, Tricia L. Nilles, MS^1^, Sean X. Leng, MD, PhD^1, 2, 4^, Joseph B. Margolick, MD, PhD^1^


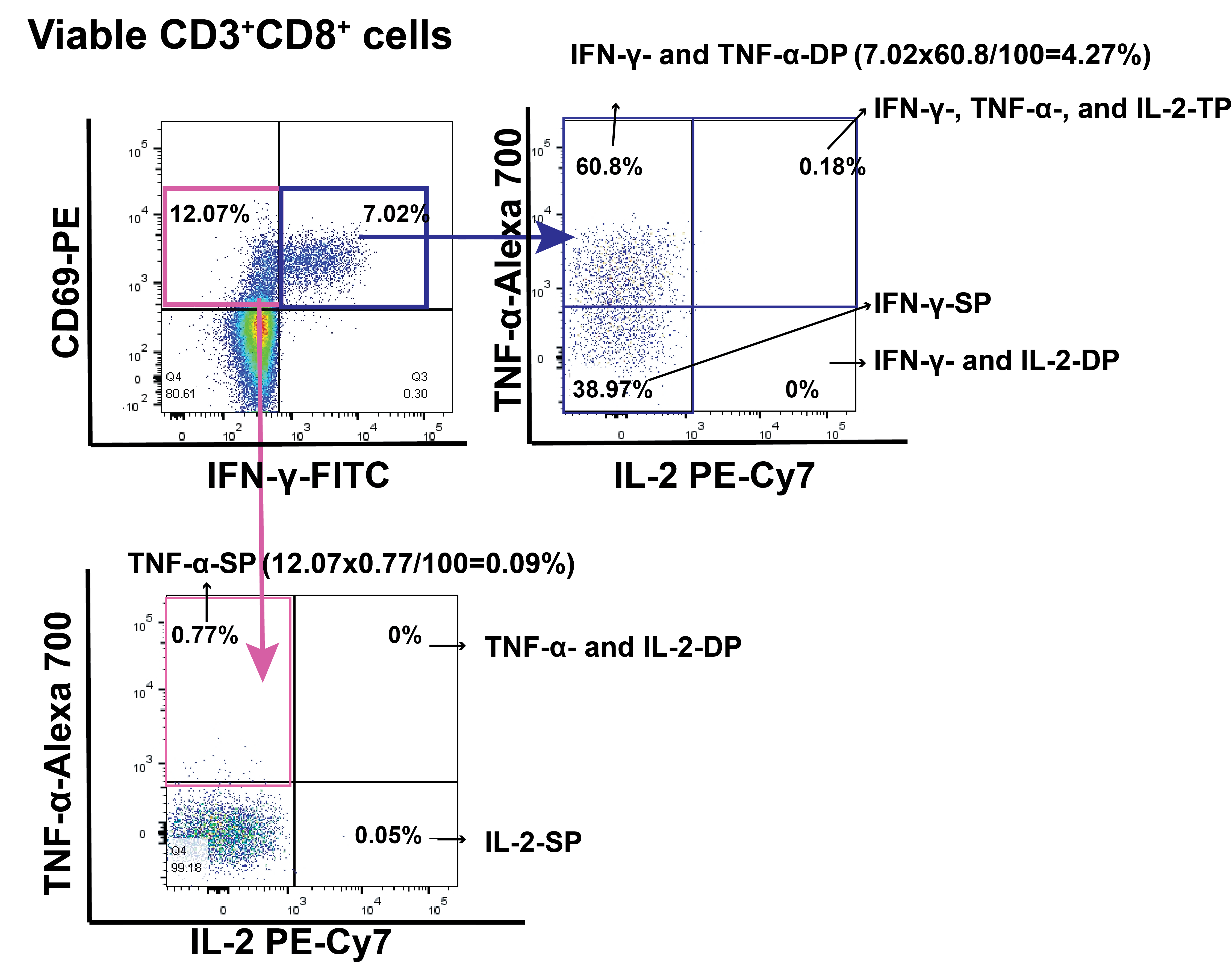


Supplementary Figure 1. Gating strategy for identifying single-, double-, and triple-cytokine-producing CD8 T cells. After identifying viable CD8 T cells via forward scatter/side scatter and expression of Aqua LIVE/DEAD dye, CD3, and CD8, production of IFN-γ, and/or TNF-α, and/or IL-2 was measured in CD69+ CD8 T cells as shown. The percentage of a given cytokine-producing CD8 T cell subset among CD8 T cells was subsequently calculated as the product of the percentage of the first gating quadrant and the proportion of the second gating quadrant used to define the subset. For example, calculation of the percentages of TNF-⍺-single-producing and of IFN-γ- and TNF-α-double-producing CD8 T cells is shown. The same gating strategy was applied to CD4 T cells.


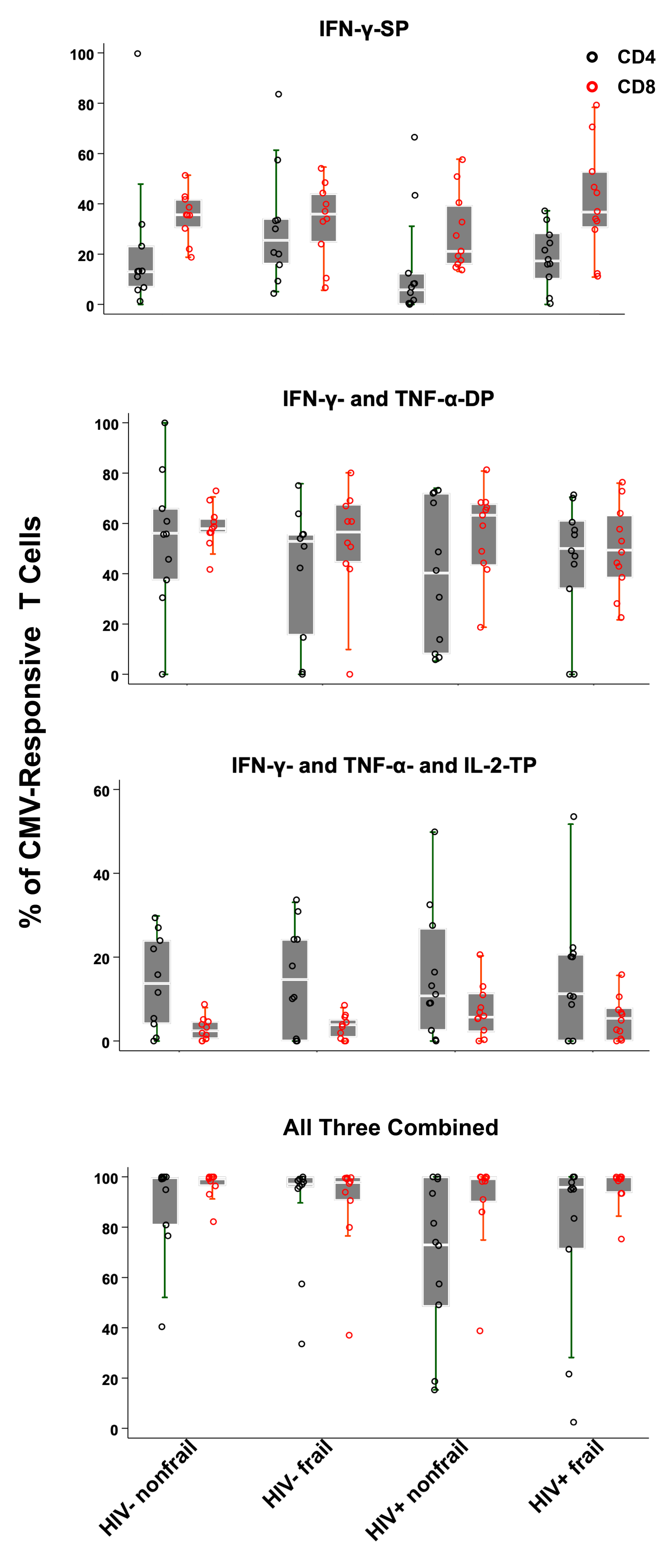


Supplementary Figure 2. Percentages of the three most common cytokine-producing phenotypes, i.e., IFN-γ-single-producing (SP), IFN-γ- and TNF-α-double-producing (DP), and IFN-γ-, TNF-α, and IL-2-triple-producing (TP), and the sums of the percentages of these three phenotypes, among total CMV-specific CD4 (black) and CD8 (red) T cells, stratified by HIV status and frailty status. Each circle represents one donor. The median, IQR, and range for each donor group are indicated by the boxplots.


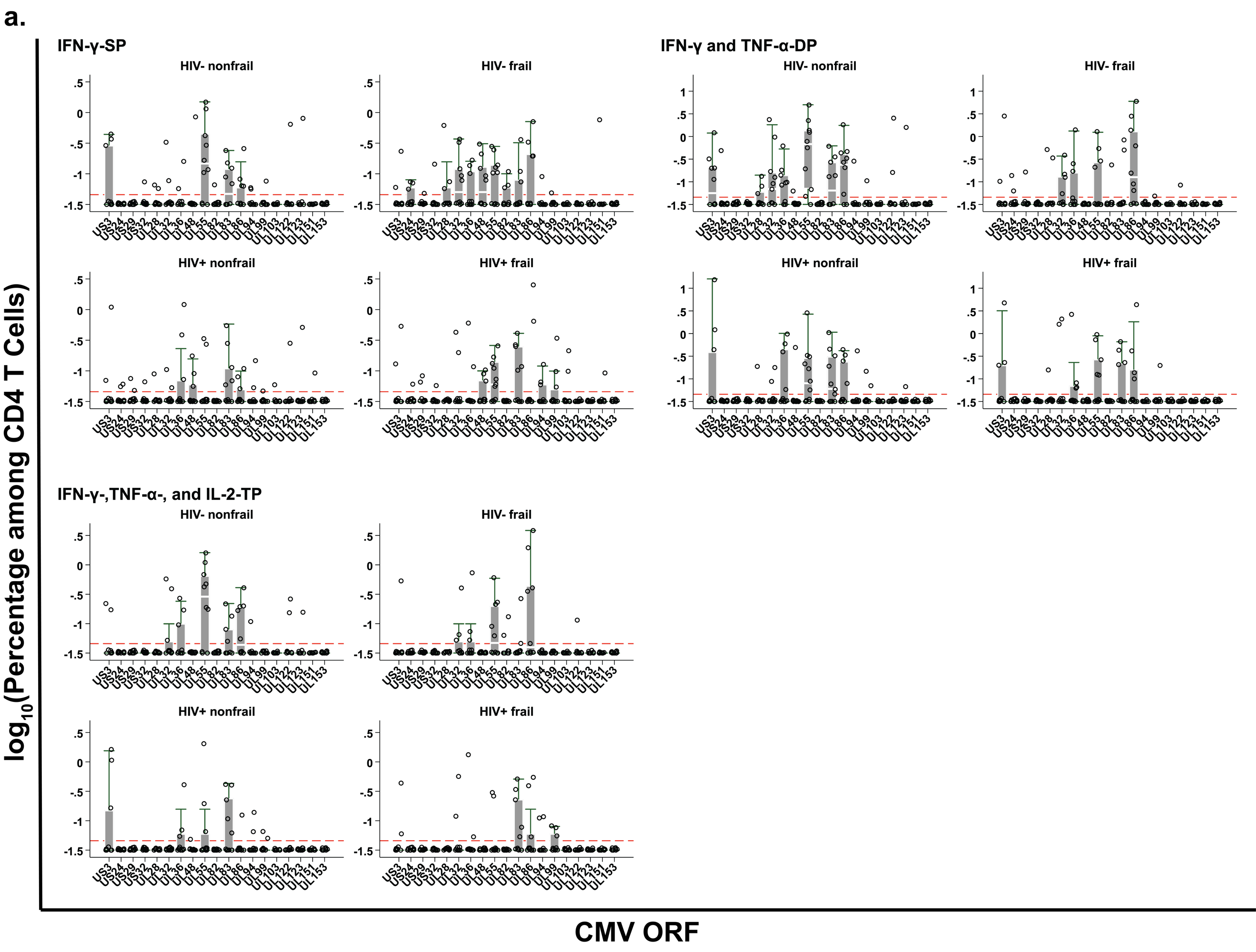


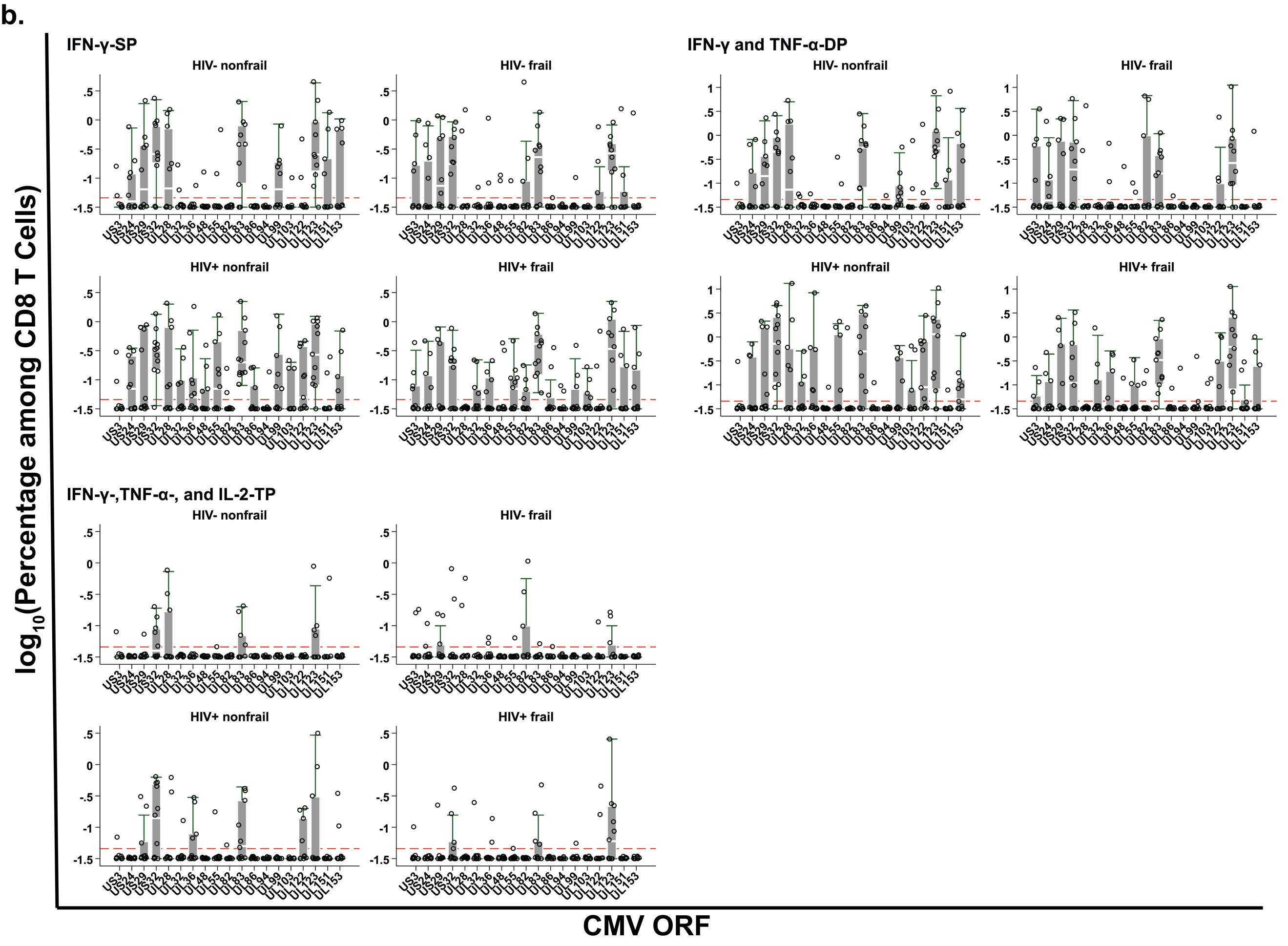


Supplementary Figure 3. Percentages of IFN-γ-SP, IFN-γ- and TNF-α-DP, and IFN-γ-, TNF-α-, and IL-2-TP generated in response to each of the 19 CMV ORFs, stratified by HIV status and frailty status, among CD4 (a) and CD8 (b) T cells. Each circle represents one donor, and the median, IQR, and range for each ORF are indicated by the boxplots. The percentages are log_10_-transformed to enhance visualization. The red dashed lines indicate the threshold of detection of CMV-responsive cells (0.05%).


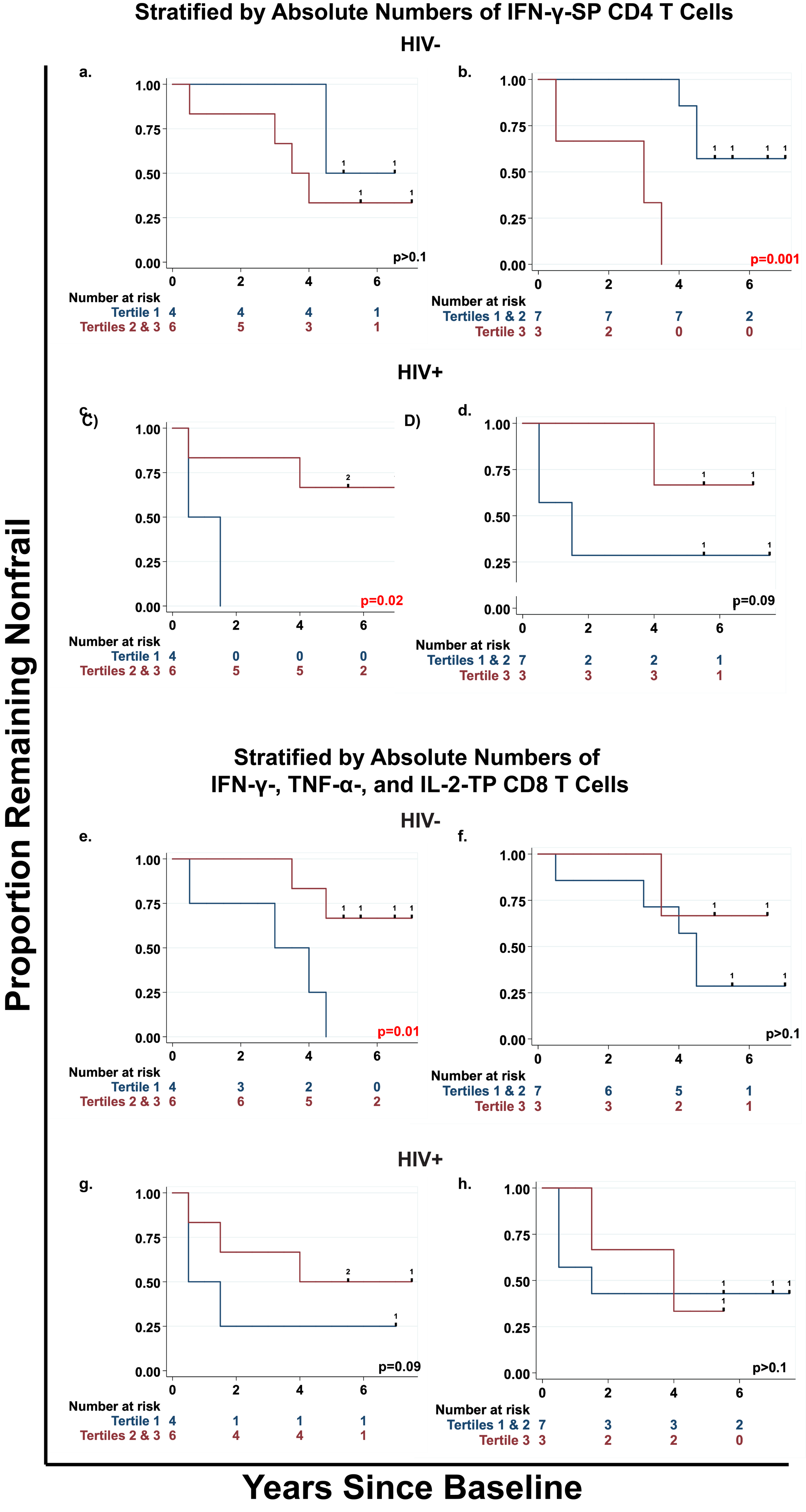


Supplementary Figure 4. Prediction of remaining nonfrail in HIV- frail men by absolute counts of IFN-γ-SP CD4 and TP CD8 subsets of CMV-responsive T cells. Kaplan-Meier survival curves (unadjusted) show the proportion remaining nonfrail among HIV- and HIV+ men, stratified by tertiles of absolute counts of IFN-γ-SP CD4 T cells (a-d) and of TP CD8 T cells (e-h). The left column shows comparisons of men in the lowest tertile of these numbers versus those in the upper two tertiles. The right column shows comparisons of men in the top tertile versus those in the lower two tertiles. Numbers at risk, p values, hash markers, and numbers on the survival curves are as described in the legend of Figure 1.


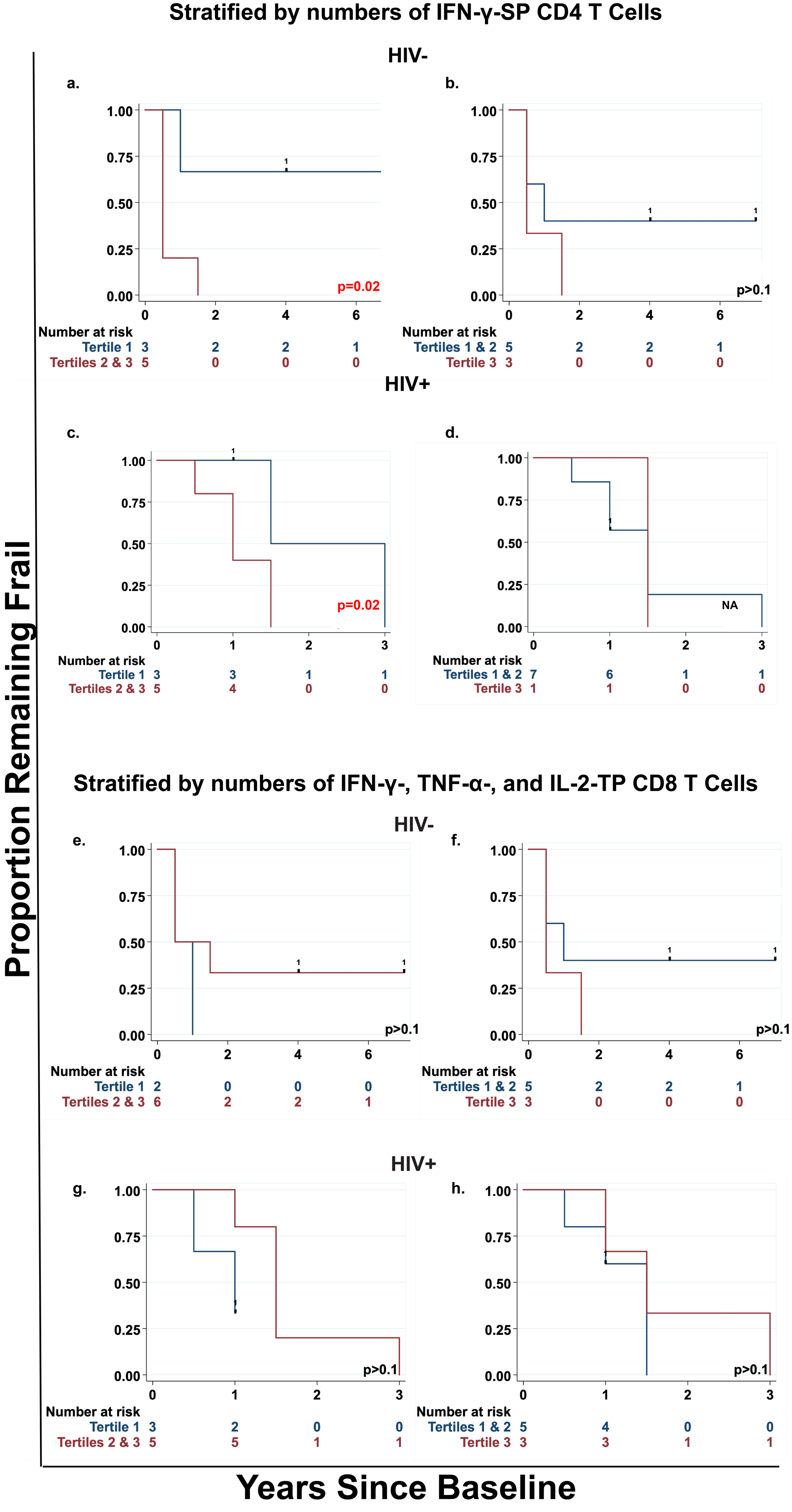


Supplementary Figure 5. Prediction of remaining frail in HIV- frail men by absolute counts of IFN-γ-SP CD4 and TP CD8 subsets of CMV-responsive T cells. Kaplan-Meier survival curves (unadjusted) show the proportion remaining frail among HIV- and HIV+ men, stratified by tertiles of absolute counts of IFN-γ-SP CD4 T cells (a-d) and of TP CD8 T cells (e-h). The left column shows comparisons of men in the lowest tertile of these numbers versus those in the upper two tertiles. The right column shows comparisons of men in the top tertile versus those in the lower two tertiles. Numbers at risk, p values, hash markers, and numbers on the survival curves are as described in the legend of Figure 1. The p value could not be determined for figure d because only one man was in the top tertile.

a.


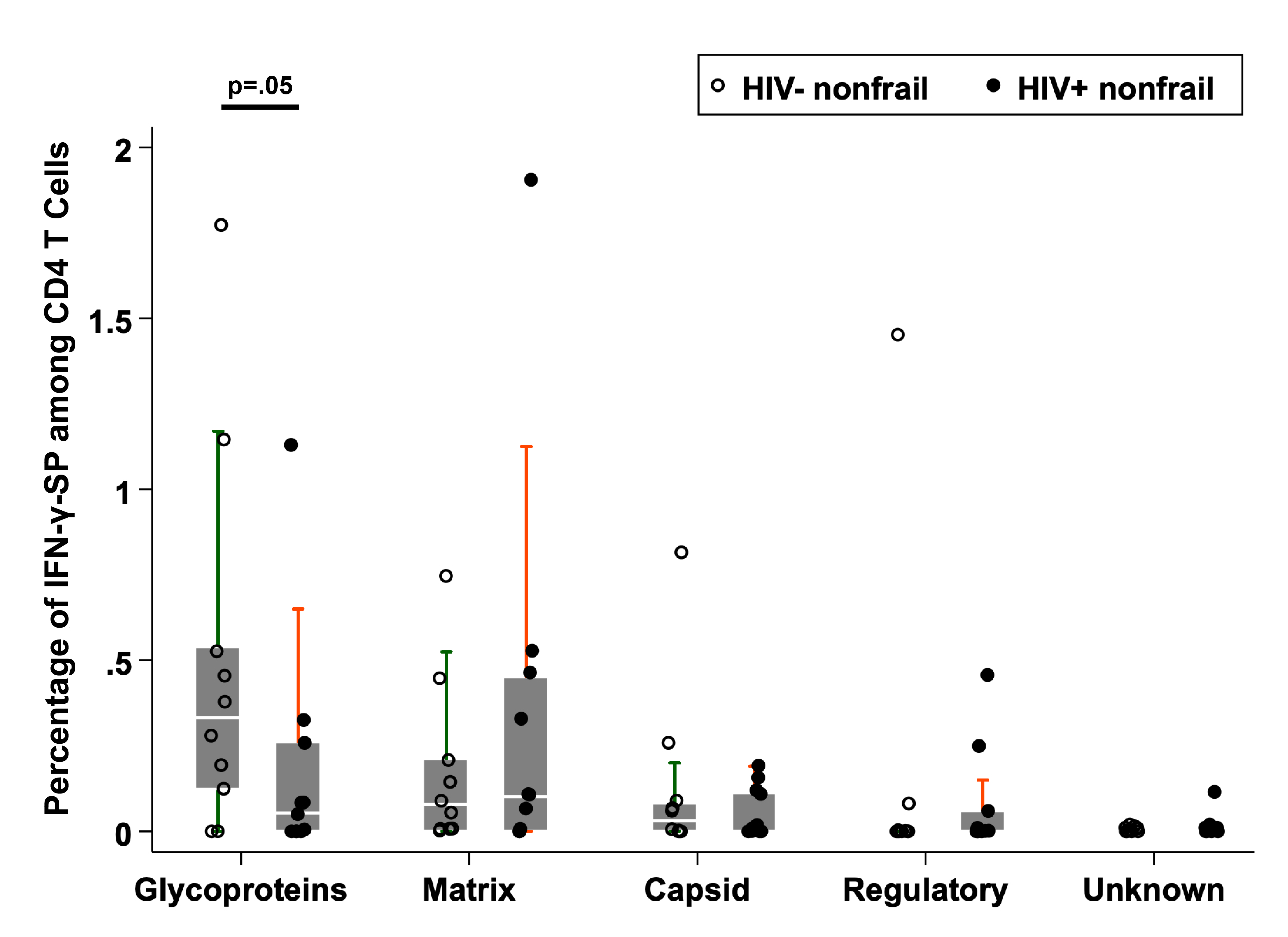


b.


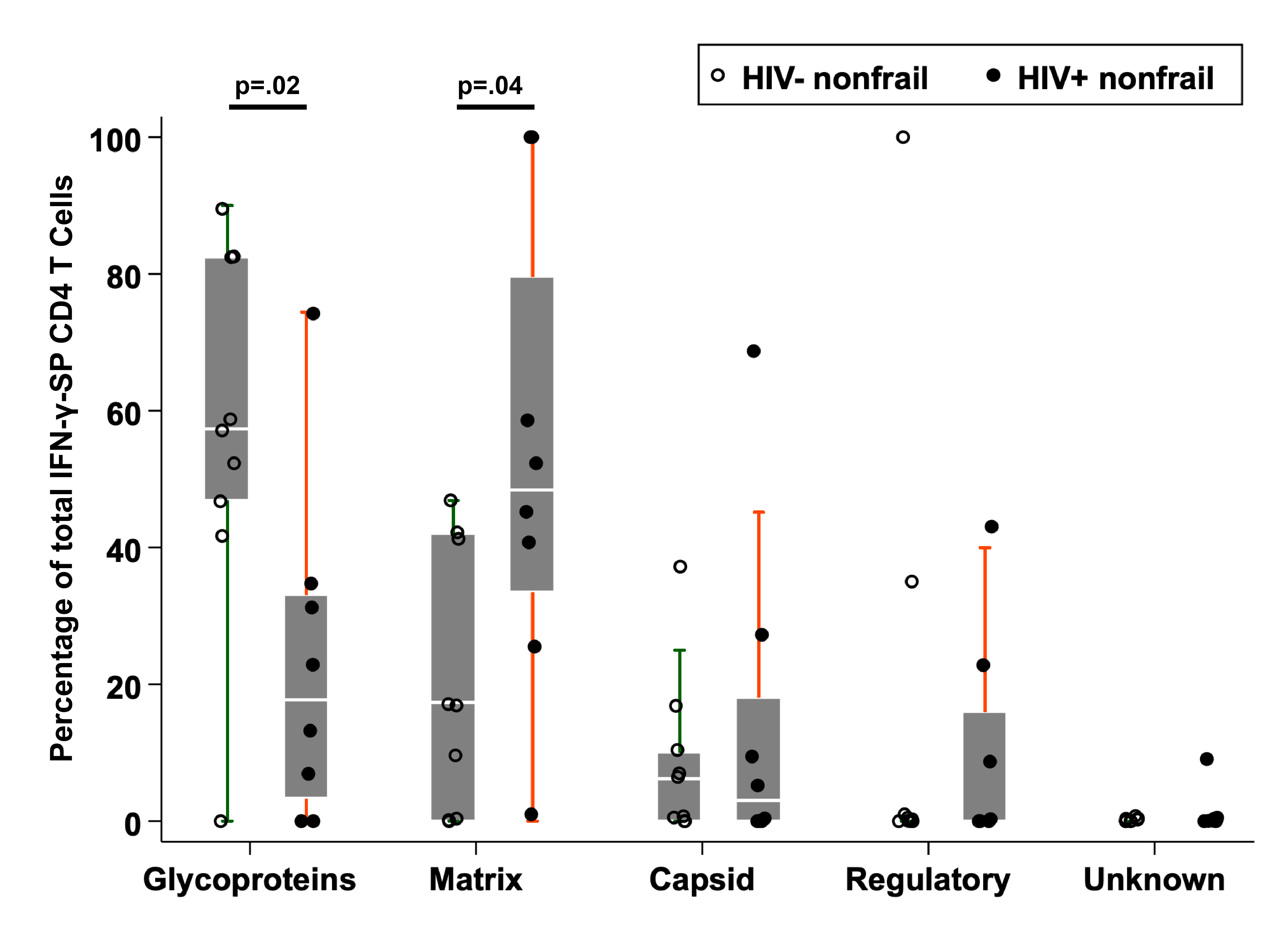


Supplementary Figure 6. Responsiveness of IFN-γ-SP CD4 T cells to CMV ORFs. Magnitude of the IFN-γ-SP CD4 T cell response to CMV open reading frames (ORFs) that were elicited by specific functional categories of CMV ORFs, expressed a) as a percentage of CD4 T cells, and b) as a percentage of the total CD4 IFN-γ-SP response. Data are shown from HIV- nonfrail men (hollow circles) and HIV+ nonfrail men (filled circles). The 19 CMV ORFs tested were categorized based on the function of their encoded proteins: glycoproteins (UL55, US3, and US29), matrix (UL32, UL36, UL82, UL83, UL94, UL99, UL103, US24, and UL28), capsid (UL48 and UL86), regulatory (UL122, UL123, and US32), and unknown (UL151 and UL153).


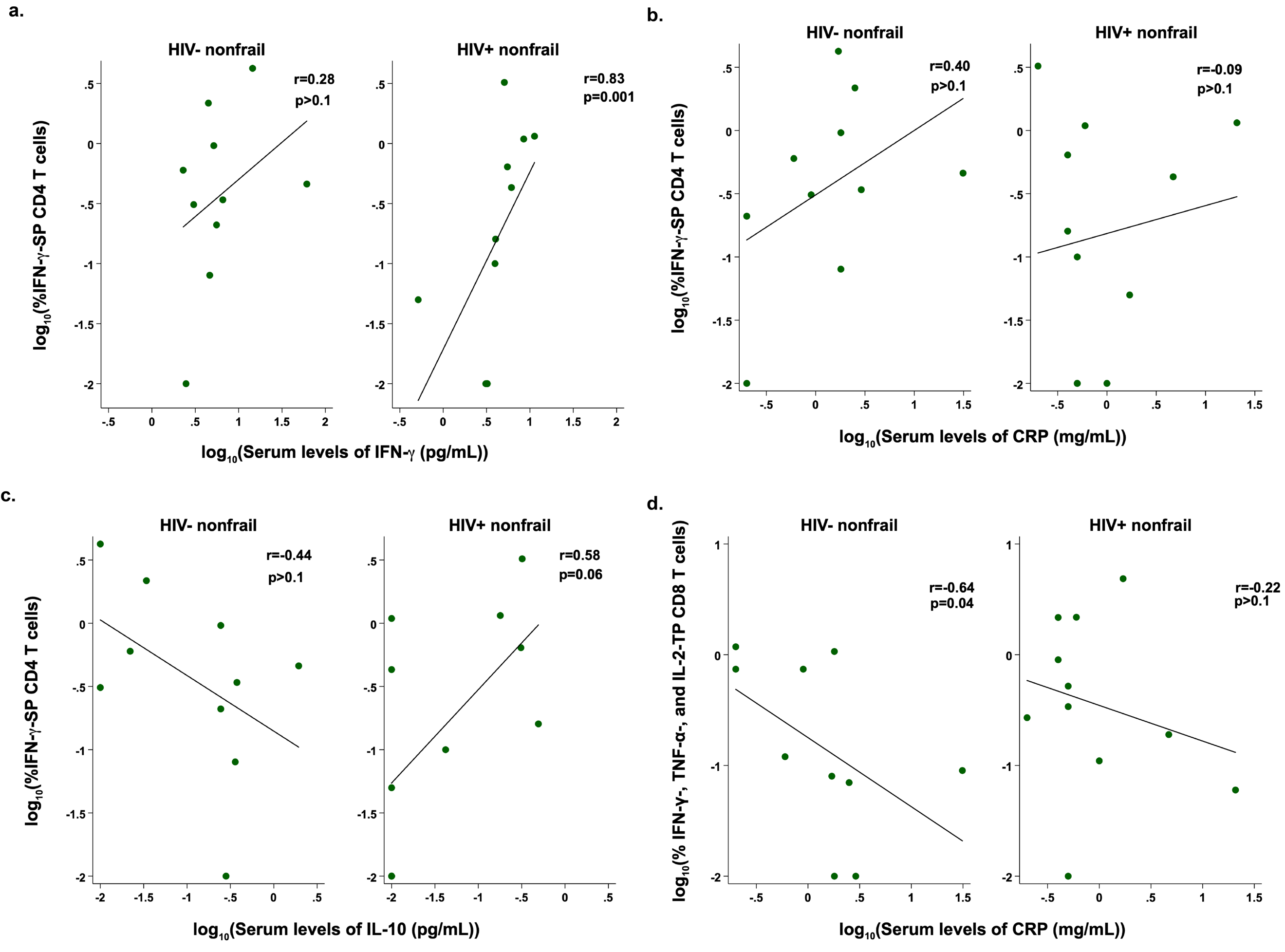


Supplementary Figure 7. Correlations between percentages of CMV-responsive T cells and serum concentrations of inflammatory markers. a-c) Correlations between percentages of CMV-specific IFN-γ-SP CD4 T cells (among total CD4 T cells) and serum levels of IFN-γ (a), CRP (b), and IL-10 (c) in HIV- and HIV+ nonfrail men. d) the correlation between percentages of IFN-𝛾-, TNF-⍺-, and IL-2-TP CD8 T cells (among total CD8 T cells) and serum levels of CRP. Each point represents one donor, and the line represents the least squared regression line. Data were log_10_-transformed, with undetectable values coded as -2. Nonparametric Spearman’s correlation coefficients are shown.


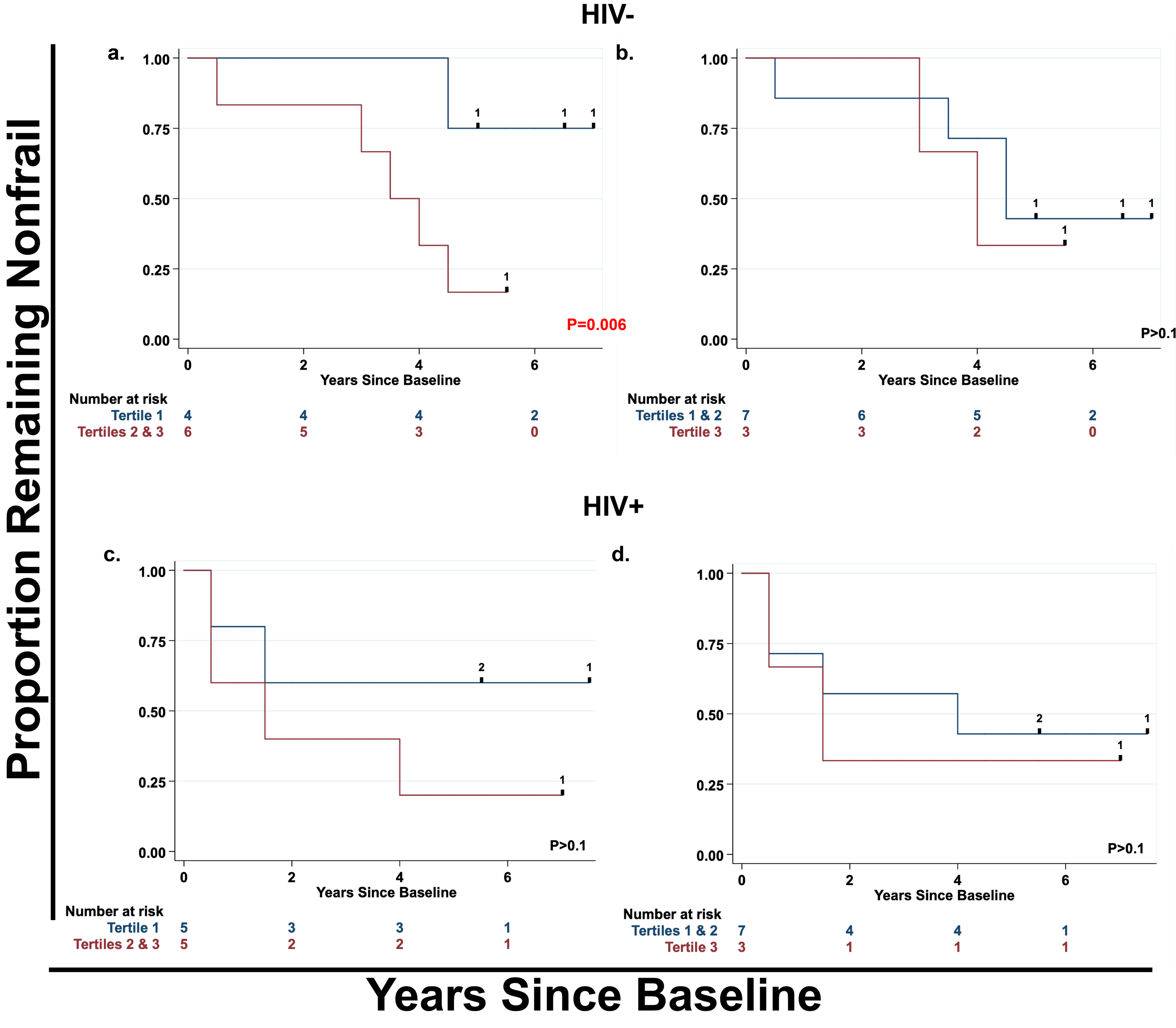


Supplementary Figure 8. Prediction of becoming frail by serum levels of CRP in HIV- and HIV+ men. Kaplan-Meier survival curves (unadjusted) show the proportion remaining nonfrail among HIV- and HIV+ men ((a-d), stratified by tertiles of serum levels of CRP. The left column compares people in the lowest tertile of these percentages versus those in the upper two tertiles among HIV- (a) and HIV+ (c) men. The right column compares people in the top tertile versus those in the lower two tertiles among HIV- (b) and HIV+ (d) men. Number at risk, p values, hash markers, and numbers on the survival curves are as described in legend of Figure 1.


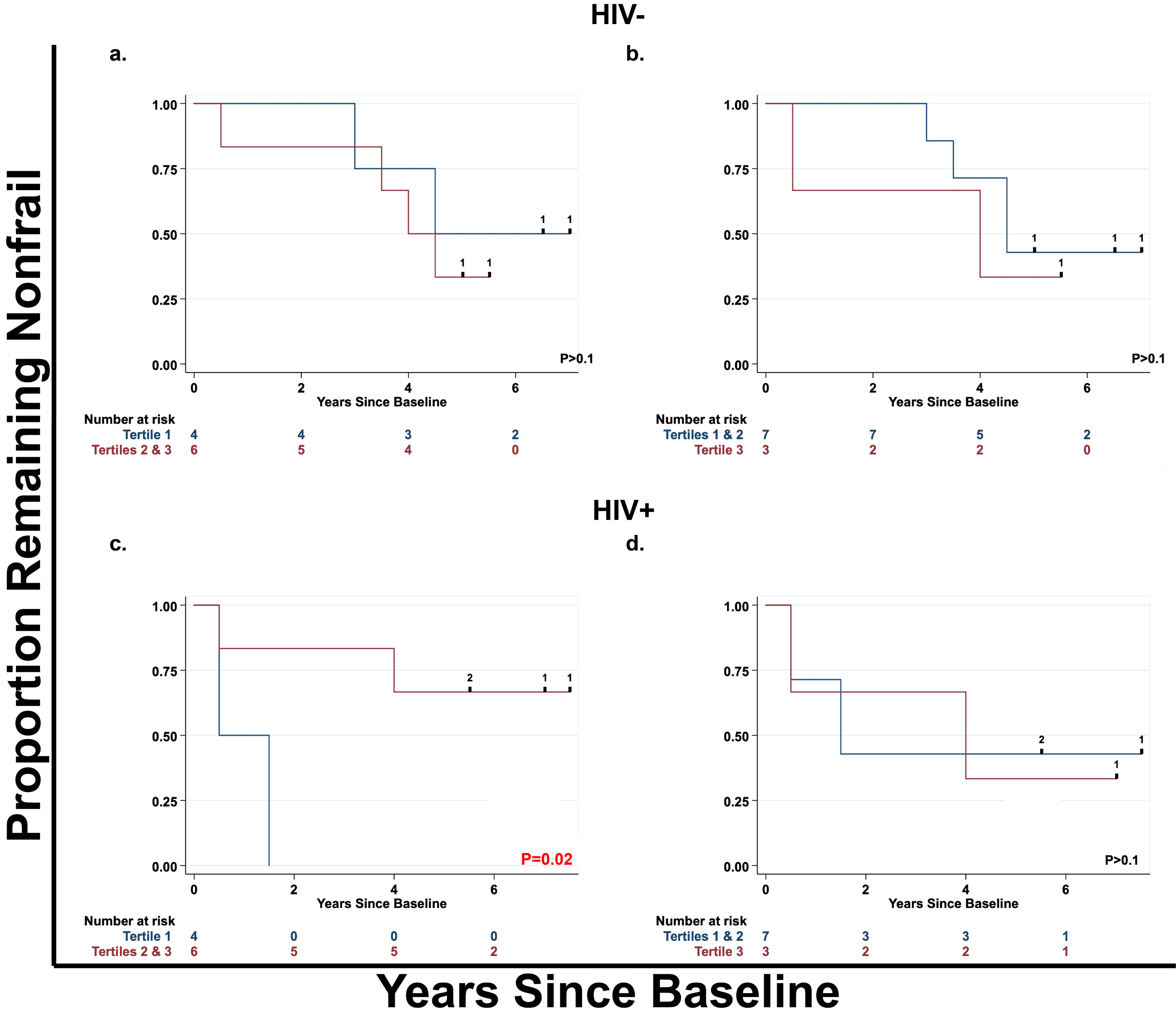


Supplementary Figure 9. Prediction of becoming frail by serum levels of IFN-γ in HIV- and HIV+ men. Kaplan-Meier survival curves (unadjusted) show the proportion remaining nonfrail among HIV- (a, b) and HIV+ men (c, d), stratified by tertiles of serum levels of IFN-γ (pg/mL). The left column shows people in the lowest tertile of these percentages versus those in the upper two tertiles among HIV- (a) and HIV+ (c) men. The right column compares people in the top tertile versus those in the lower two tertiles among HIV- (b) and HIV+ (d) men. Number at risk, p values, hash markers, and numbers on the survival curves are as described in legend of Figure 1.


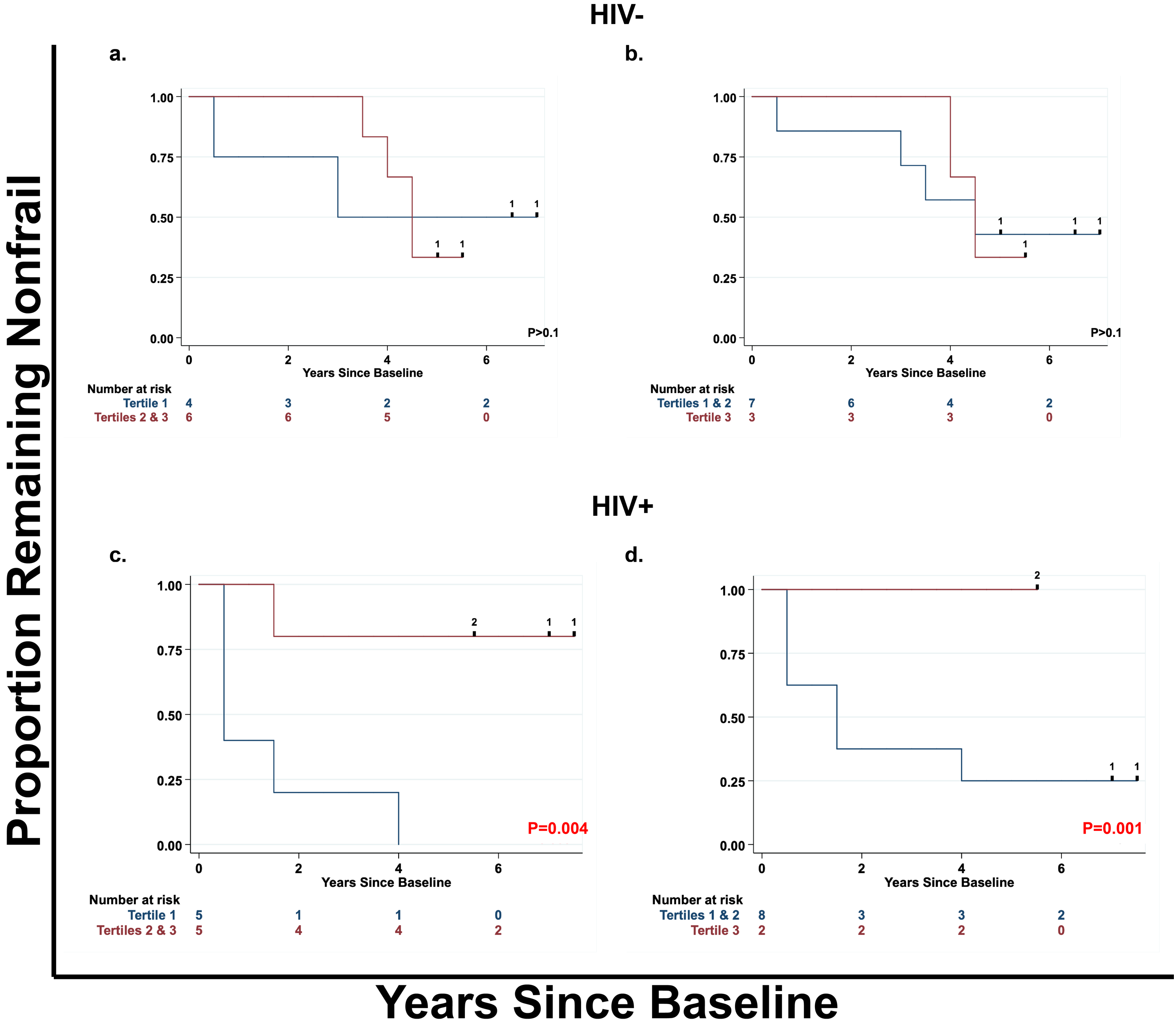


Supplementary Figure 10. Prediction of becoming frail by serum levels of IL-10 in HIV- and HIV+ men. Kaplan-Meier survival curves (unadjusted) show the proportion remaining nonfrail among HIV- and HIV+ men ((a-d), stratified by tertiles of serum levels of IL-10 (pg/mL). The left column shows people in the lowest tertile of these percentages versus those in the upper two tertiles among HIV- (a) and HIV+ (c) men. The right column compares people in the top tertile versus those in the lower two tertiles among HIV- (b) and HIV+ (d) men. Number at risk, p values, hash markers, and numbers on the survival curves are as described in legend of Figure 1.


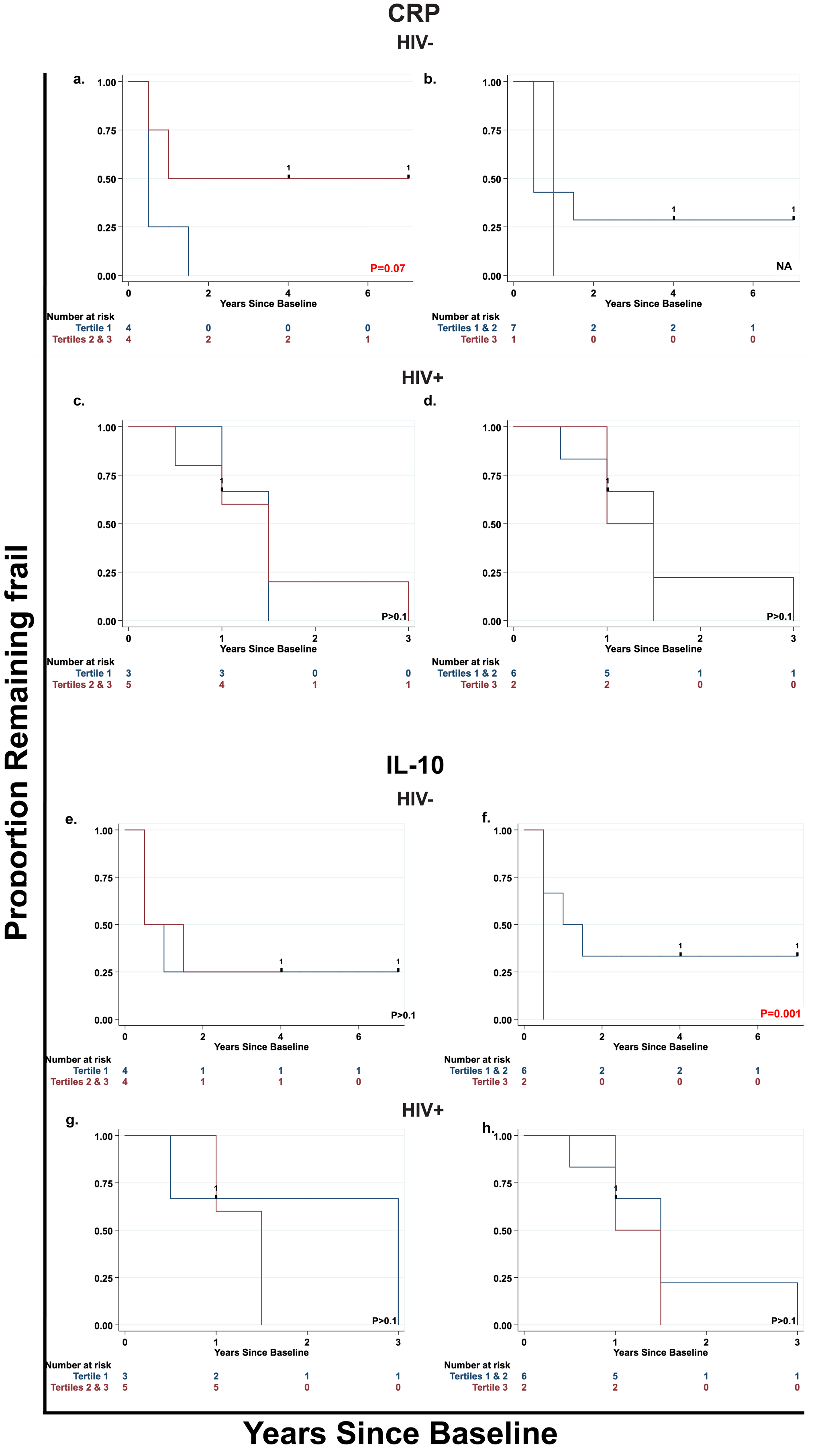


Supplementary Figure 11. Prediction of remaining frail in HIV- men by serum levels of CRP and IL-10. Kaplan-Meier survival curves (unadjusted) show the proportion remaining frail among HIV- and HIV+ men, stratified by tertiles of percentages of CRP (a-d) and of IL-10 (e-h). The left column shows men in the lowest tertile of these percentages versus those in the upper two tertiles among HIV- (a and e) and HIV+ (c and g) men. The right column compares men in the top tertile versus those in the lower two tertiles among HIV- (b and f) and HIV+ (d and h) men. Number at risk, p values, hash markers, and numbers on the survival curves are as described in legend of Figure 1. The P value could not be determined for figure b because only one man was in the top tertile of CRP.

Supplementary Table 1. Human CMV open reading frames (ORFs) of the peptide pools tested in this study ^a^

| CMV ORF | Amino Acid  Length | ∑ # Peptides  Mixed | Product  Function^b^ |
| --- | --- | --- | --- |
| UL 28 | 379 | 74 | Matrix |
| UL 32 (pp150) | 1048 | 208 | Matrix |
| UL 36 | 476 | 94 | Matrix |
| UL 48 | 2241 | 448 | Capsid |
| UL 55 (gB) | 906 | 180 | Glycoproteins |
| UL 82 (pp71) | 558 | 110 | Matrix |
| UL 83 (pp65) | 561 | 111 | Matrix |
| UL 86 | 1370 | 272 | Capsid |
| UL 94 | 344 | 67 | Matrix |
| UL 99 (pp28) | 190 | 36 | Matrix |
| UL103 | 249 | 47 | Matrix |
| UL122 (IE-2) | 580 | 114 | Regulatory |
| UL123 (IE-1) | 419 | 82 | Regulatory |
| UL151 | 336 | 65 | Unknown |
| UL153 | 277 | 53 | Unknown |
| US 3 | 186 | 36 | Glycoproteins |
| US24 | 500 | 98 | Matrix |
| US29 | 462 | 90 | Glycoproteins |
| US32 | 183 | 34 | Regulatory |

^a^ This table is adapted from Li, et al. J. Infect. Dis. 2014, 210, 400–404 (1). Detailed information, including amino acid sequence, can be found in Sylwester, et al. J. Exp. Med. 2005; 202:673-85 (2).

^b^ The functions of UL28, UL103, US3, and US32 are updated according to references (3) and (4).

Supplementary Table 2. Cut-off values of percentages and absolute counts of IFN-γ-SP CD4 T Cells and IFN-γ-, TNF-α-, and IL-2-TP CD8 T cells for defining tertiles

|  | IFN-γ-SP CD4 T Cells | | | | IFN-γ-, TNF-α-, and IL-2-TP CD8 T cells | | | |
| --- | --- | --- | --- | --- | --- | --- | --- | --- |
| Group | Tertile 1 vs Tertiles 2 & 3 | | Tertile 1& 2 vs Tertile 3 | | Tertile 1 vs Tertiles 2 & 3 | | Tertile 1& 2 vs Tertile 3 | |
|  | % | Absolute Counts ( cells/μL) | % | Absolute Counts ( cells/μL) | % | Absolute Counts ( cells/μL) | % | Absolute Counts ( cells/μL) |
| HIV- nonfrail | 0.3 | 4.4 | 1.0 | 8.8 | 0.1 | 0.5 | 1.1 | 3.5 |
| HIV+ nonfrail | 0.1 | 0.7 | 1.1 | 5.7 | 0.3 | 2.9 | 2.2 | 14.9 |
| HIV- frail | 0.5 | 4.5 | 1.3 | 13.5 | 0.3 | 1.0 | 0.6 | 4.6 |
| HIV+ frail | 0.1 | 1.4 | 1.9 | 10.9 | 0.2 | 2.2 | 0.7 | 9.8 |

Supplementary Table 3. Demographic Characteristics of the Men Studied (N = 42)

| Variable | Total | | HIV Status | | | | |
| --- | --- | --- | --- | --- | --- | --- | --- |
|  | (N = 42) | | HIV^+^ (n = 22) | | HIV^−^ (n = 20) | | P* |
|  | Median | IQR | Median | IQR | Median | IQR |  |
| Age, y | 61 | 9 | 58 | 10 | 63 | 7 | .0310 |
| Education, y | 16 | 4 | 16 | 2 | 16 | 4 | .5082 |
| CD4 /μL | 1038 | 551 | 715 | 612 | 1139 | 301 | .0044 |
| CD8 /μL | 800 | 721 | 1167 | 648 | 604 | 217 | .0002 |
| HCV^a^ | 7 |  | 6 |  | 1 |  | .096 |
| Ethnicity | | | | | | | |
| White | 33 |  | 16 |  | 17 |  | .46 |
| Black | 9 |  | 6 |  | 3 |  |  |
| Smoking | | | | | | | |
| Current | 9 |  | 7 |  | 2 |  | .25 |
| Former | 18 |  | 8 |  | 10 |  |  |
| Never smoked | 15 |  | 7 |  | 8 |  |  |
| Depressive symptoms | 14 |  | 7 |  | 7 |  | 1.0 |
| Hypertension | 28 |  | 13 |  | 15 |  | .34 |
| Diabetes | 9 |  | 3 |  | 6 |  | .44 |
| Dyslipidemia | 31 |  | 16 |  | 15 |  | 1.0 |
| Kidney disease | 3 |  | 3 |  |  |  | .23 |
| Liver disease | 6 |  | 5 |  | 1 |  | .18 |
| Nadir CD4 /μL |  |  | 288 | 157 |  |  |  |
| CD4/CD8 ratio |  |  | 0.82 | 0.47 |  |  |  |
| Years of cART |  |  | 17 | 5 |  |  |  |
| Years untreated |  |  | 12 | 7 |  |  |  |
| HIV RNA (copies/mL) |  |  | 40 | n/a |  |  |  |
| History of AIDS | 4 |  | 4 |  |  |  |  |

Note. Abbreviations: cART, combination antiretroviral therapy; IQR, interquartile range; n/a, not applicable.

^a^ Hepatitis C was defined as either a positive enzyme immunosorbent assay (EIA)

for anti-hepatitis C virus (HCV) antibody on plasma or serum, or detection of HCV RNA in plasma.

^*^P value comparing HIV^+^ and HIV^−^ subjects.

Reproduced with permission from (5)

Supplementary Table 4. Concordance of categorization by tertiles using percentages and numbers of IFN-γ-SP CD4 T Cells (a) and IFN-γ-,TNF-α-, and IL-2-TP CD8 T cells (b).

a.

|  | Numbers of IFN-γ-SP CD4 T cells (/μL) | | | | | |
| --- | --- | --- | --- | --- | --- | --- |
| % IFN-γ-SP CD4 T cells | Nonfrail | | | Frail | | |
|  | Tertile 1 | Tertiles 2 & 3 | Kappa* | Tertile 1 | Tertiles 2 & 3 | Kappa |
| Tertile 1 | 8 | 0 | 1 | 6 | 2 | 0.68 |
| Tertiles 2 & 3 | 0 | 13 |  | 1 | 11 |  |
|  |  |  |  |  |  |  |
|  | Tertiles 1 & 2 | Tertile 3 | Kappa | Tertiles 1 & 2 | Tertile 3 | Kappa |
| Tertiles 1 & 2 | 15 | 0 | 1 | 14 | 1 | 0.88 |
| Tertile 3 | 0 | 6 |  | 0 | 5 |  |

b.

|  | Numbers of IFN-γ and TNF-⍺ and IL-2-TP CD8 T cells (/μL) | | | | | |
| --- | --- | --- | --- | --- | --- | --- |
| % IFN-γ-, TNF-⍺-, and IL-2-TP  CD8 T cells | Nonfrail | | | Frail | | |
|  | Tertile 1 | Tertiles 2 & 3 | Kappa | Tertile 1 | Tertiles 2 & 3 | Kappa |
| Tertile 1 | 8 | 0 | 1 | 6 | 2 | 0.68 |
| Tertiles 2 & 3 | 0 | 13 |  | 1 | 11 |  |
|  |  |  |  |  |  |  |
|  | Tertiles 1 & 2 | Tertile 3 | Kappa | Tertiles 1 & 2 | Tertile 3 | Kappa |
| Tertiles 1 & 2 | 15 | 1 | 0.88 | 14 | 1 | 0.88 |
| Tertile 3 | 0 | 5 |  | 0 | 5 |  |

The numbers for one HIV- frail man are missing.

* Kappa statistic(6)

Supplementary Table 5. Significant predictors of adverse frailty-related outcomes by polyfunctional T cell subsets.

|  | Faster Onset of Frailty | | Greater Maintenance of Frailty | |
| --- | --- | --- | --- | --- |
| T cell Subset | HIV- | HIV+ | HIV- | HIV+ |
| IFN-γ-SP CD4^a^ | Higher | Lower | Lower | Lower |
| TP CD8^b^ | Lower | NS^c^ | Lower | NS |

^a^ CD4 T cells producing only IFN-γ

^b^ CD8 T cells producing IFN-𝛾-, TNF-⍺-, and IL-2

^c^ not significant

Supplementary Table 6. IFN-γ-only CD4 T cell responses to peptide pools spanning CMV UL55, US3, and US29 open reading frames, among HIV- and HIV+ nonfrail men.

|  | UL55 | | US3 | | US29 | |
| --- | --- | --- | --- | --- | --- | --- |
|  | HIV- | HIV+ | HIV- | HIV+ | HIV- | HIV+ |
|  | n=10 | n=11 | n=10 | n=11 | n=10 | n=11 |
| Men who had positive IFN-γ-SP CD4 T cell response (n (%)) | **7 (70)^a^** | **2 (18)** | 3 (30) | 2 (18) | 0 (0) | 2 (18) |
| Percentages of IFN-γ-SP CD4 T cells among total CD4 T cells (median (IQR) %) | **0.15 (0.45)** | **0.00 (0.00)** | 0.00 (0.29) | 0.00 (0.00) | 0.00 (0.00) | 0.00 (0.00) |

^a^ bold values indicate significant difference (p<0.05) between HIV- and HIV+ nonfrail men.

Supplementary Table 7. Summary of prediction of onset and maintenance of frailty by serum levels of inflammatory markers

|  | Faster Onset of Frailty | | | | Greater Maintenance of Frailty | | | |
| --- | --- | --- | --- | --- | --- | --- | --- | --- |
|  |  |  |  |  |  |  |  |  |
|  |  |  |  |  |  |  |  |  |
|  | HIV- | | HIV+ | | HIV- | | HIV+ | |
| Inflammatory Markers | Tertile 1 vs Tertiles 2 and 3 | Tertiles 1 and 2 vs Tertile 3 | Tertile 1 vs Tertiles 2 and 3 | Tertiles 1 and 2 vs Tertile 3 | Tertile 1 vs Tertiles 2 and 3 | Tertiles 1 and 2 vs Tertile 3 | Tertile 1 vs Tertiles 2 and 3 | Tertiles 1 and 2 vs Tertile 3 |
| CRP | Higher (p=0.006) | NS^a^ | NS | NS | Higher (p=0.07) | NS | NS | NS |
| IL-10 | NS | NS | Lower  (p=0.004) | Lower (p=0.001) | NS | Lower | NS | NS |
| IFN-𝛾 | NS | NS | Lower (p=0.02) | NS | NS | NS | NS | NS |

^a^ No significant association

Supplementary Table 8. Proportion of follow-up visits with manifestation of frailty among HIV- and HIV+ men who were frail at baseline, stratified by tertiles of serum levels of CRP, IL-10, and IFN-γ

|  | HIV- frail (n=8) | | | | | | HIV+ frail (n=8) | | | | | |
| --- | --- | --- | --- | --- | --- | --- | --- | --- | --- | --- | --- | --- |
|  | Tertile 1 | Tertiles 2 & 3 | p-value | Tertiles 1 & 2 | Tertile 3 | p-value | Tertile 1 | Tertiles 2 & 3 | p-value | Tertiles 1 & 2 | Tertile 3 | p-value |
| CRP | 55.0 (22.9)* | 91.7 (39.6) | 0.1 | 60.0 (62.5) | 83.3 ( 0.0) | 0.5 | 80.0 (85.7) | 37.5 (31.4) | 0.7 | 58.8 (76.7) | 44.3 (31.4) | 0.7 |
| IL-10 | 60.4 (58.6) | 62.1 (27.1) | 0.7 | 73.8 (62.5) | 55.0 (10.0) | 0.5 | 93.3 (83.3) | 37.5 (31.4) | 0.3 | 70.0 (76.7) | 33.0 ( 8.9) | 0.5 |
| IFN-𝛾 | 60.0 (71.4) | 64.3 (33.3) | 0.8 | 62.1 (45.8) | 75.0 (50.0) | 0.6 | 70.0 (20.0) | 33.0 (76.7) | 0.5 | 70.0 (76.7) | 33.0 ( 8.9) | 0.5 |

*Median (IQR)

References

1. Li H, Margolick JB, Bream JH, Nilles TL, Langan S, Bui HT, et al. Heterogeneity of CD4+ and CD8+ T-cell responses to cytomegalovirus in HIV-infected and HIV-uninfected men who have sex with men. J Infect Dis. 2014;210(3):400–4.

2. Sylwester AW, Mitchell BL, Edgar JB, Taormina C, Pelte C, Ruchti F, et al. Broadly targeted human cytomegalovirus-specific CD4+ and CD8+ T cells dominate the memory compartments of exposed subjects. J Exp Med. 2005;202(5):673.

3. Salsman J, Zimmerman N, Chen T, Domagala M, Frappier L. Genome-Wide Screen of Three Herpesviruses for Protein Subcellular Localization and Alteration of PML Nuclear Bodies. PLoS Pathog [Internet]. 2008 Jul 11 [cited 2019 Jul 29];4(7). Available from: https://www.ncbi.nlm.nih.gov/pmc/articles/PMC2438612/

4. Mocarski ES, Shenk T, Griffiths PD, Pass RF. Cytomegaloviruses. In: Fields Virology. 6th ed. Philadelphia, PA: Wolters Kluwer/Lippincott Williams & Wilkins Health; 2013. p. 1970.

5. Margolick JB, Bream JH, Nilles TL, Li H, Langan SJ, Deng S, et al. Relationship Between T-Cell Responses to CMV, Markers of Inflammation, and Frailty in HIV-uninfected and HIV-infected Men in the Multicenter AIDS Cohort Study. J Infect Dis. 2018 Jun 20;218(2):249–58.

6. McHugh ML. Interrater reliability: the kappa statistic. Biochem Medica. 2012 Oct 15;22(3):276–82.
